# Supplementary material for: Artificial kagome lattices of Shockley surface states patterned by halogen hydrogen-bonded organic frameworks
Source: Nat Commun. 2024 Apr 6;15:2969. doi: 10.1038/s41467-024-47367-5 (PMC10998891; doi:10.1038/s41467-024-47367-5)
Supplement: Supplementary file 1 — Supplementary information [file 41467_2024_47367_MOESM1_ESM.pdf]

Supplementary Information for

Artificial kagome lattices of Shockley surface states patterned by halogen hydrogen-bonded organic frameworks

Ruoting Yin[1,#], Xiang Zhu[1,#], Qiang Fu[1,2], Tianyi Hu[1], Lingyun Wan[1], Yingying Wu[1], Yifan Liang[1], Zhengya Wang[1], Zhen-Lin Qiu[3], Yuan-Zhi Tan[3], Chuanxu Ma[1,2,*], Shijing Tan[1,2], Wei Hu[1,2], Bin Li[1,2], Z. F. Wang[1,2], Jinlong Yang[1,2] and Bing Wang[1,2,*]

[1]Hefei National Research Center for Physical Sciences at the Microscale and Synergetic Innovation Center of Quantum Information & Quantum Physics, New Cornerstone Science Laboratory, University of Science and Technology of China, Hefei, Anhui 230026, China

[2]Hefei National Laboratory, University of Science and Technology of China, Hefei 230088, China

[3]Collaborative Innovation Center of Chemistry for Energy Materials, State Key Laboratory for Physical Chemistry of Solid Surfaces, and Department of Chemistry, College of Chemistry and Chemical Engineering, Xiamen University, 361005 Xiamen, China

[#]These authors contributed equally: Ruoting Yin and Xiang Zhu.

*Corresponding authors: cxma85@ustc.edu.cn (C.M.); bwang@ustc.edu.cn (B.W.)

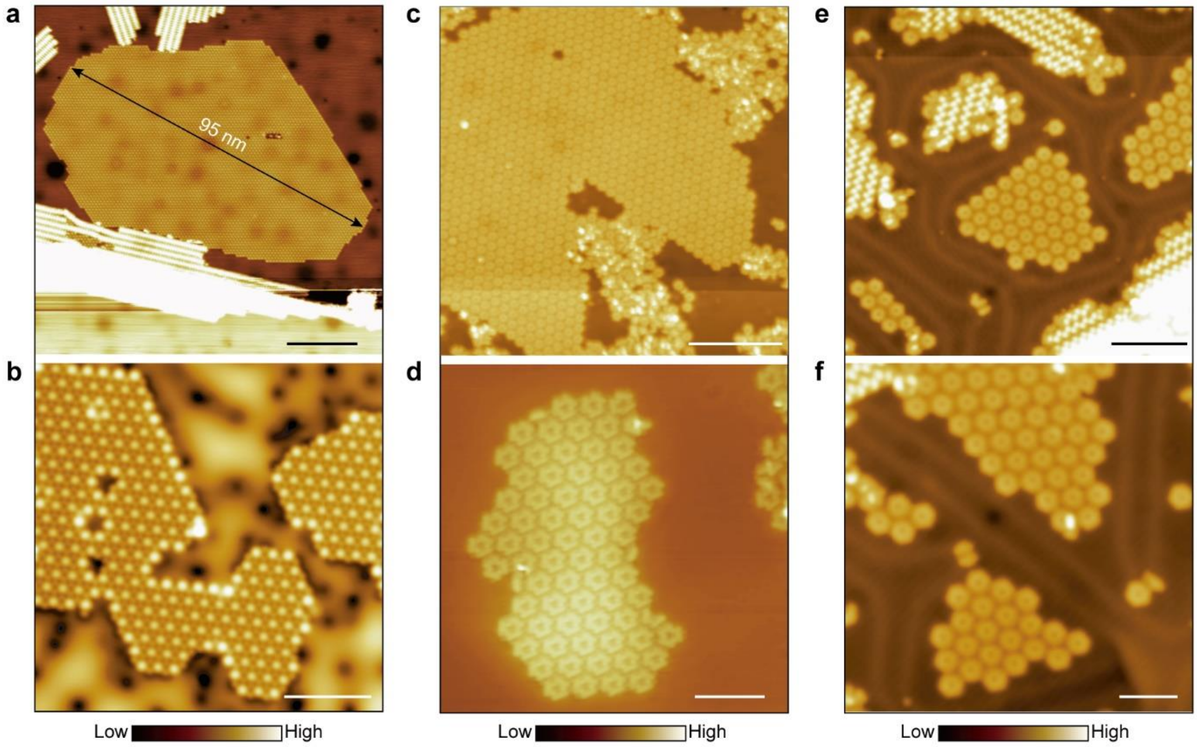

Supplementary Fig. 1 | STM topographic images of the XHOFs. **a**, **b**, The benzene/Br/Ag(111) superlattice. **c**,**d**, The M-C66/Br/Ag(111) superlattice. **e**,**f**, The C66/Br/Au(111) superlattice. STM imaging conditions: **a**, V_s = 100 mV, I_t = 200 pA; **b**, V_s = 100 mV, I_t = 200 pA; **c**, V_s = −2.00 V, I_t = 10 pA; **d**, V_s = 1.00 V, I_t = 10 pA; **e**, V_s = −2.00 V, I_t = 10 pA; **f**, V_s = −2.00 V, I_t = 10 pA. Scale bars: **a**, 20 nm; **b**, 5 nm; **c**, 20 nm; **d**, 5 nm; **e**, 10 nm; **f**, 5 nm.

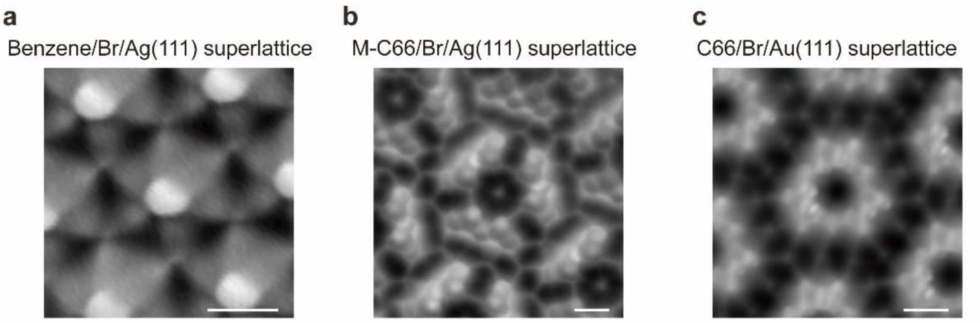

Supplementary Fig. 2 | The images corresponding the Fig. 2d,i,n without the superimposed structures. **a**, The benzene/Br/Ag(111) superlattice, **b**, the M-C66/Br/Ag(111) superlattice, and **c**, the C66/Br/Au(111) superlattice. Bright dot-like features representing Br atoms can be seen. All nc-AFM images were acquired with a CO-functionalized tip, with tip heights $\Delta z = -20$ (**a**), −5 (**b**), and −30 pm (**c**), respectively, with respect to the setpoint condition $V_s = -0.8$ V, $I_t = 10$ pA on molecules. Scale bars: **a**, 0.5 nm; **b**, 1 nm; **c**, 1 nm.

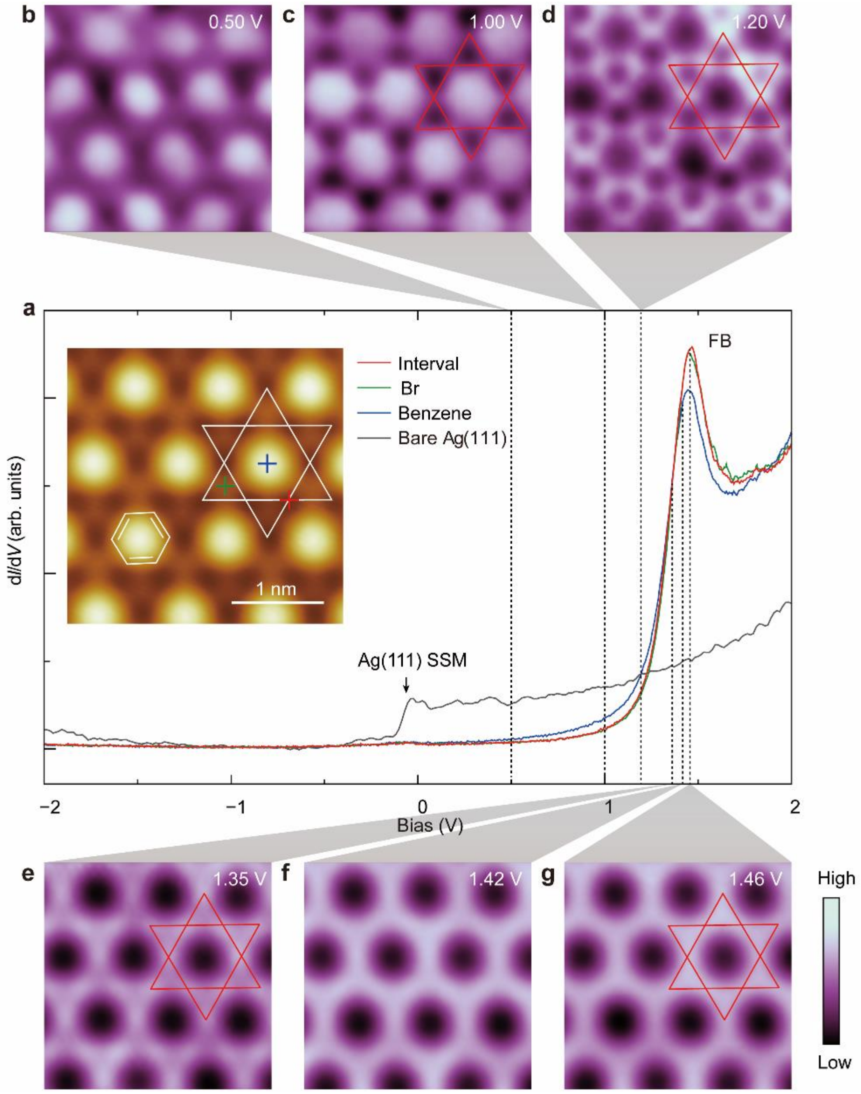

Supplementary Fig. 3 | Electronic characterizations of the benzene/Br/Ag(111) superlattice. a, Same d*I*/d*V* spectra of the benzene/Br/Ag(111) superlattice as those in Fig. 4a without vertical shift. **b−g**, d*I*/d*V* maps obtained at energies indicated in **a**. STM imaging conditions: **a**, V_s = 1.00 V, I_t = 50 pA. d*I*/d*V* measurement parameters: **a**, V_s = 2.00 V, I_t = 50 pA; **b−g**, I_t = 50 pA. Scale bars: 1 nm.

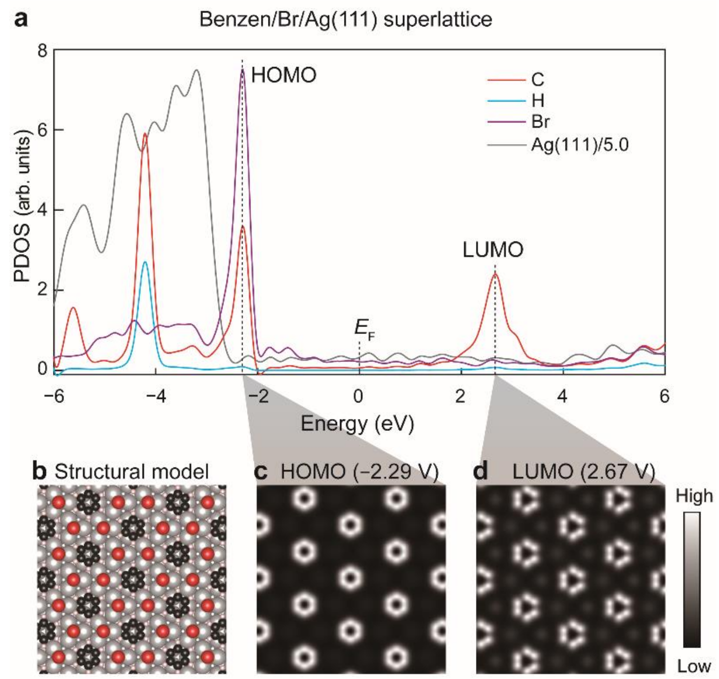

Supplementary Fig. 4 | DFT-calculated partial densities of states (PDOSs) and LDOS maps of the benzene/Br/Ag(111) superlattice. **a**, PDOSs, **b**, structural model, and **c**,**d,** simulated LDOS maps at the marked energies.

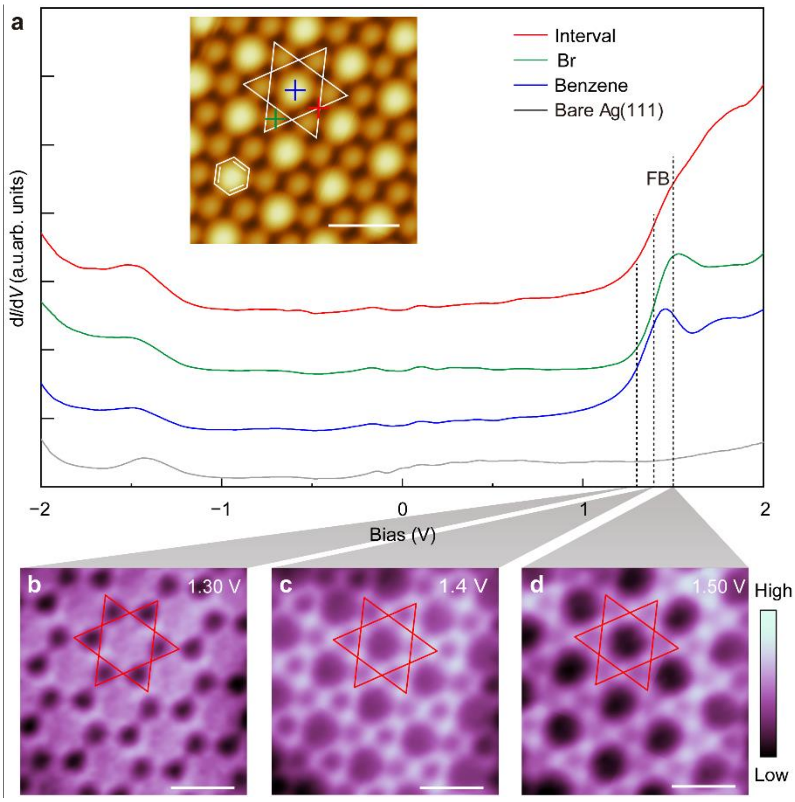

Supplementary Fig. 5 | Electronic characterizations of the benzene/Br/Ag(111) superlattice with a different tip. a, dI/dV spectra of the benzene/Br/Ag(111) superlattice. **b**−**d**, dI/dV maps obtained at energies indicated in **a**. STM imaging conditions: **a**, V_s = 100 mV, I_t = 50 pA; dI/dV measurement parameters: **a**, V_s = −2.50 V, I_t = 10 pA; **b**−**d**, I_t = 500 pA. Scale bars: 1 nm.

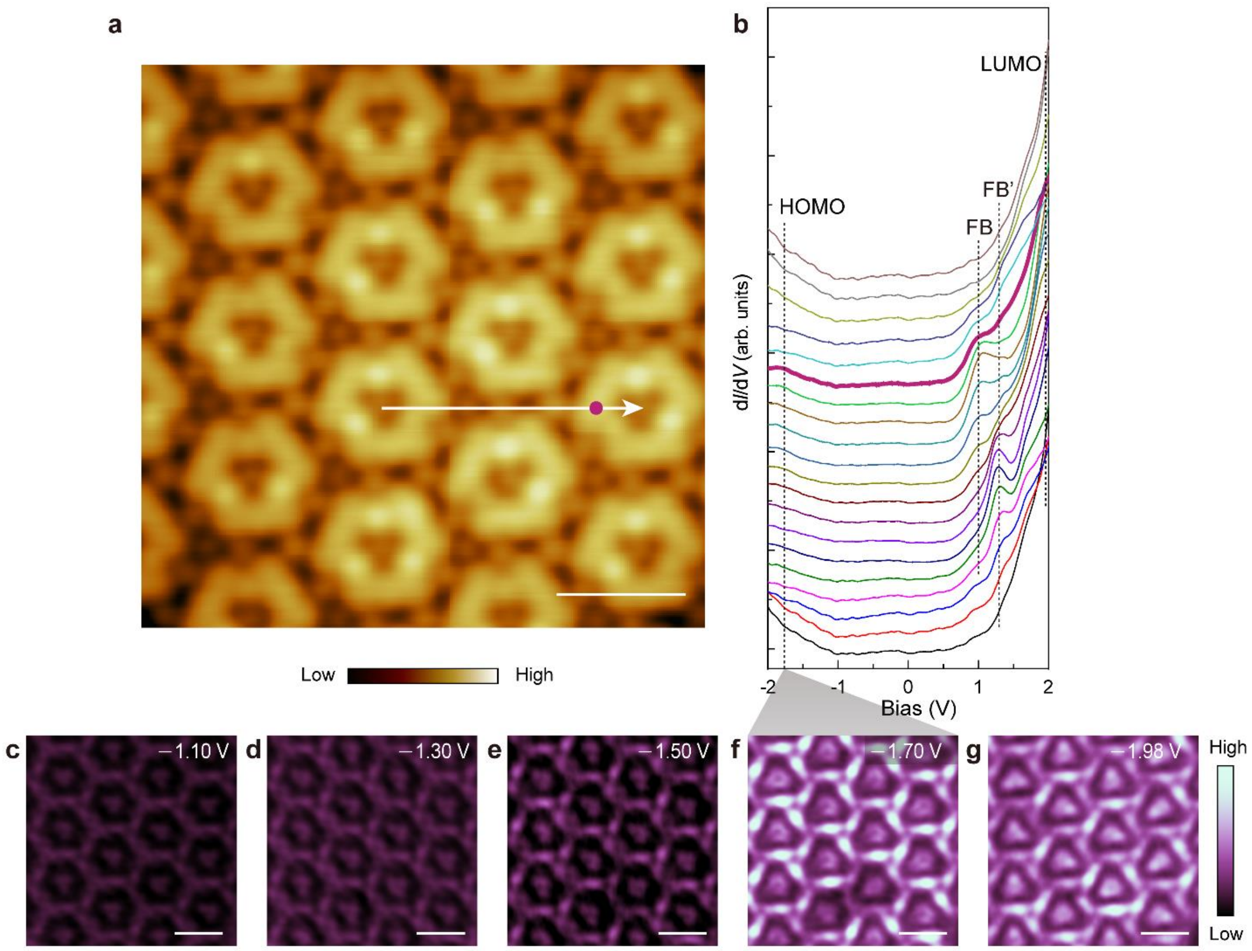

Supplementary Fig. 6 | Electronic characterization of the M-C66/Br/Ag(111) superlattice. a, STM topographic image of the M-C66/Br/Ag(111) superlattice. **b**, d*I*/d*V* spectra acquired along the arrowed line in **a**, with the bold purple curve taken on the M-C66, as marked by a purple dot in **a**. Spectra were shifted vertically from bottom to up for clarity. **c**−**g**, d*I*/d*V* maps acquired within the same area as **a**, at the marked energy in each figure. Same colour bar is used for **c**−**g**. STM imaging conditions: **a**, V_s = −130 mV, I_t = 50 pA. d*I*/d*V* measurement parameters: **b**, V_s = 2.00 V, I_t = 100 pA; **c**−**g**, I_t = 50 pA. Scale bars: 2 nm.

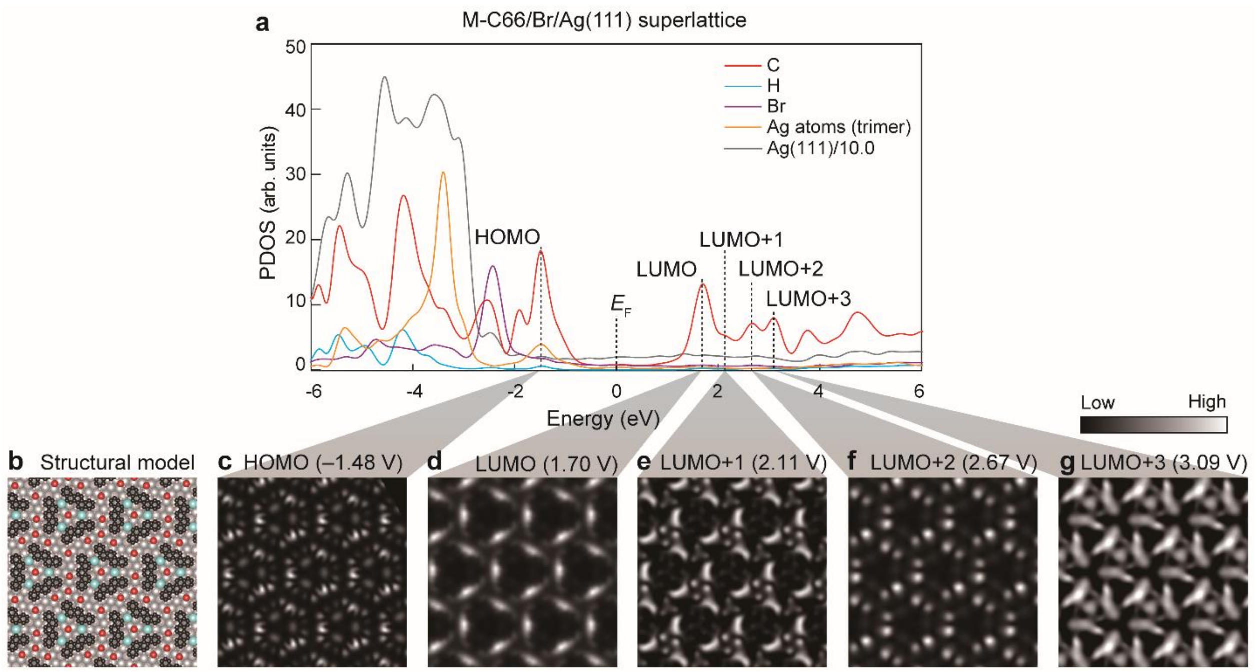

Supplementary Fig. 7 | DFT-calculated PDOSs and LDOS maps of the M-C66/Br/Ag(111) superlattice. **a**, PDOSs, **b**, structural model, and **c**−**g**, simulated LDOS maps at the marked energies.

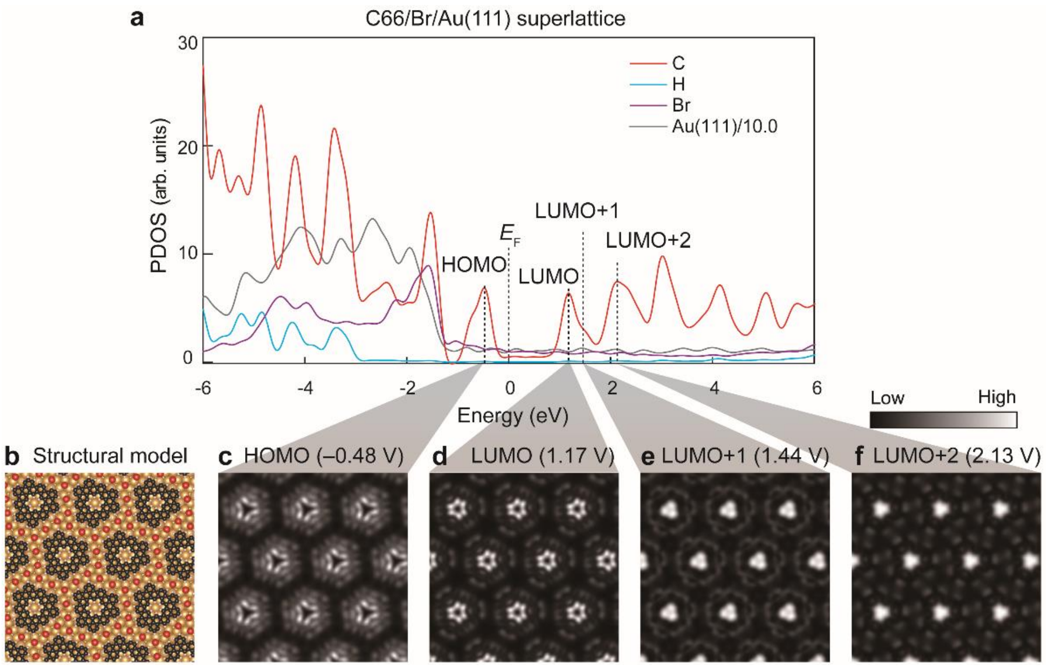

Supplementary Fig. 8 | DFT-calculated PDOSs and LDOS maps of the C66/Br/Au(111) superlattice. **a**, PDOSs, **b**, structural model, and **c**−**f**, simulated LDOS maps at the marked energies.

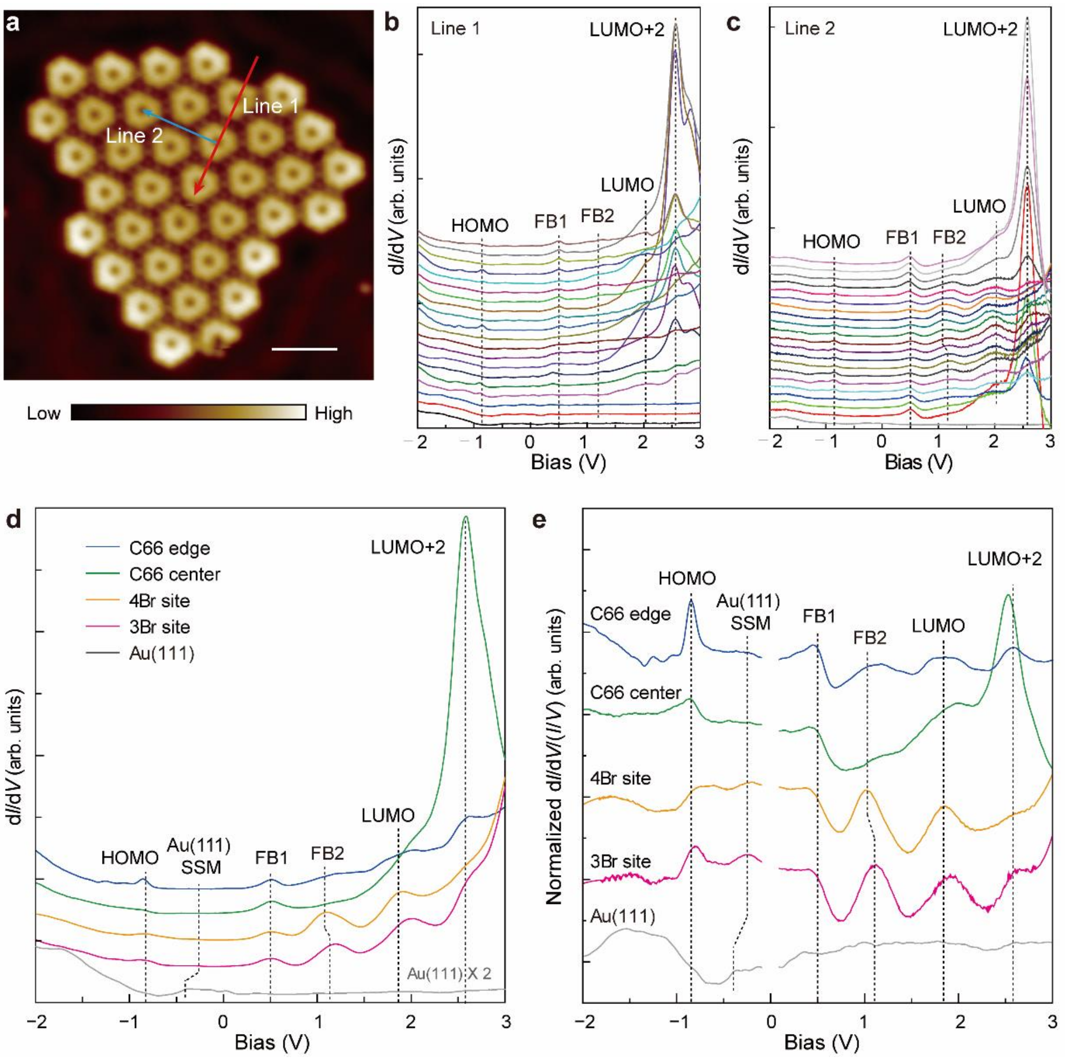

Supplementary Fig. 9 | Site-dependent d*I*/d*V* spectra in the C66/Br/Au(111) superlattice. a, STM topographic image of the C66/Br/Au(111) superlattice. Scale bar: 3 nm. **b**,**c**, d*I*/d*V* spectra acquired along the arrowed lines indicated in **a**, respectively. Spectra were shifted vertically from bottom to up for clarity. **d,** d*I*/d*V* spectra and **e,** the corresponding normalized (d*I*/d*V*)/(*I*/*V*) spectra, same as Fig. 5a in the main text, acquired at several representative positions in the C66/Br/Au(111) superlattice. STM imaging conditions: $V_s = -2.00$ V, $I_t = 10$ pA. d*I*/d*V* measurement parameters: $V_s = -2.00$ V, $I_t = 10$ pA.

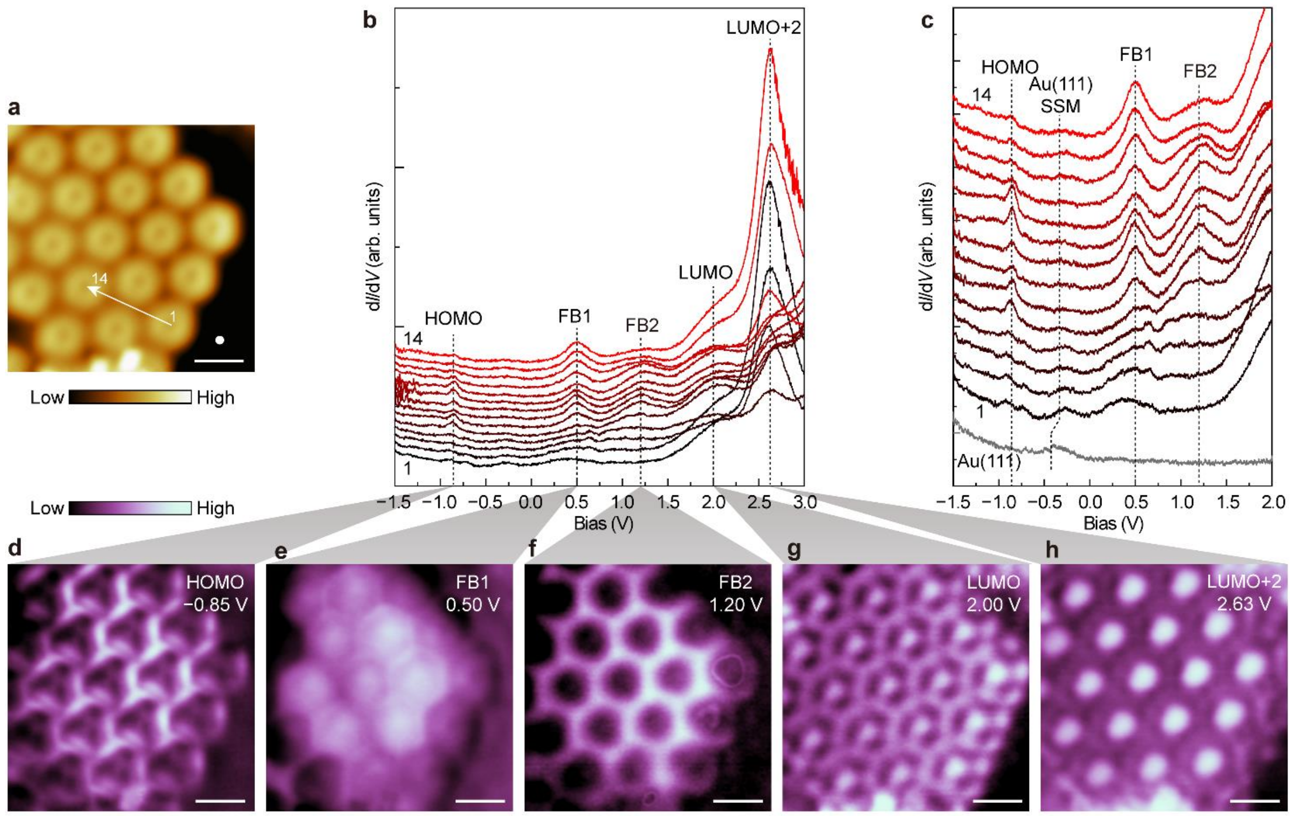

Supplementary Fig. 10 | Electronic characterization of another C66/Br/Au(111) superlattice. a, STM topographic image of the C66/Br/Au(111) island. **b**,**c**, Fourteen dI/dV spectra taken along the arrowed white line in **a**, within different energy windows. In **c**, one reference spectrum (gray) acquired at the white dot marked bare Au(111) surface in **a** is also given, showing the typical Au(111) SSM around −450 mV, which displays the upshift in the superlattice. **d**−**f**, dI/dV maps taken at energies of HOMO (**d**), FB1 (**e**), FB2 (**f**), LUMO (**g**), and LUMO+2 (**h**), as indicated in **b**. STM imaging conditions: **a**, V_s = −0.85 V, I_t =10 pA. dI/dV measurement parameters: **b**,**c**, V_s = −2.00 V, I_t =10 pA; **d**−**f**, I_t = 10 pA. Scale bars: 2 nm.

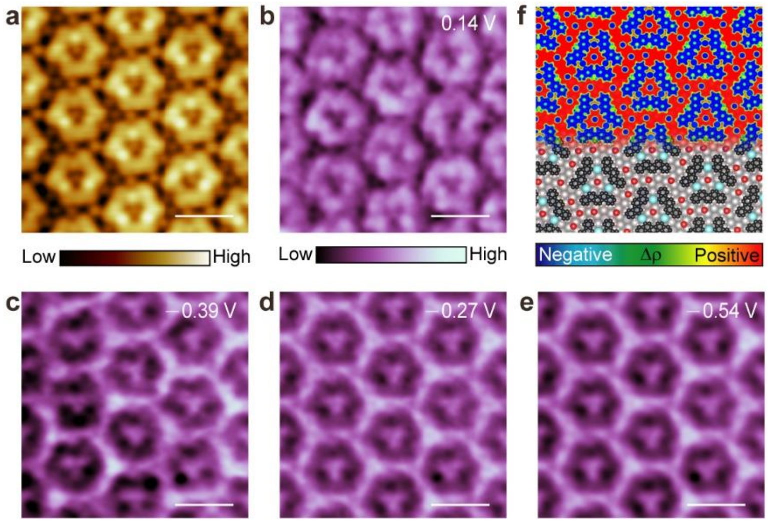

Supplementary Fig. 11 | Observation of charge order in the M-C66/Br/Ag(111) superlattice. a, STM topographic image of the M-C66/Br/Ag(111). **b**−**e**, dI/dV maps acquired within the same area as **a** at energies above (**b**) and below (**c**−**e**) the Fermi level, respectively, as marked in each figure. **f**, Simulated electrostatic potential of the M-C66/Br/Ag(111) superlattice corresponding to the same area as **a**−**e**, with the structural model superimposed at the bottom. The dI/dV map acquired at the positive bias (**b**) showing enhanced LDOS patterns well resemble the negative charge accumulation regions in the electrostatic potential map (**f**), while those acquired at the negative biases (**c**−**e**) well resemble the positive charge accumulation regions in the electrostatic potential map (**f**), consistent with the existence of charge order. STM imaging conditions: **a**, $V_s = -130$ mV, I_t=50 pA. dI/dV measurement parameters: **b**−**e**, I_t = 50 pA. Scale bars: 2 nm.

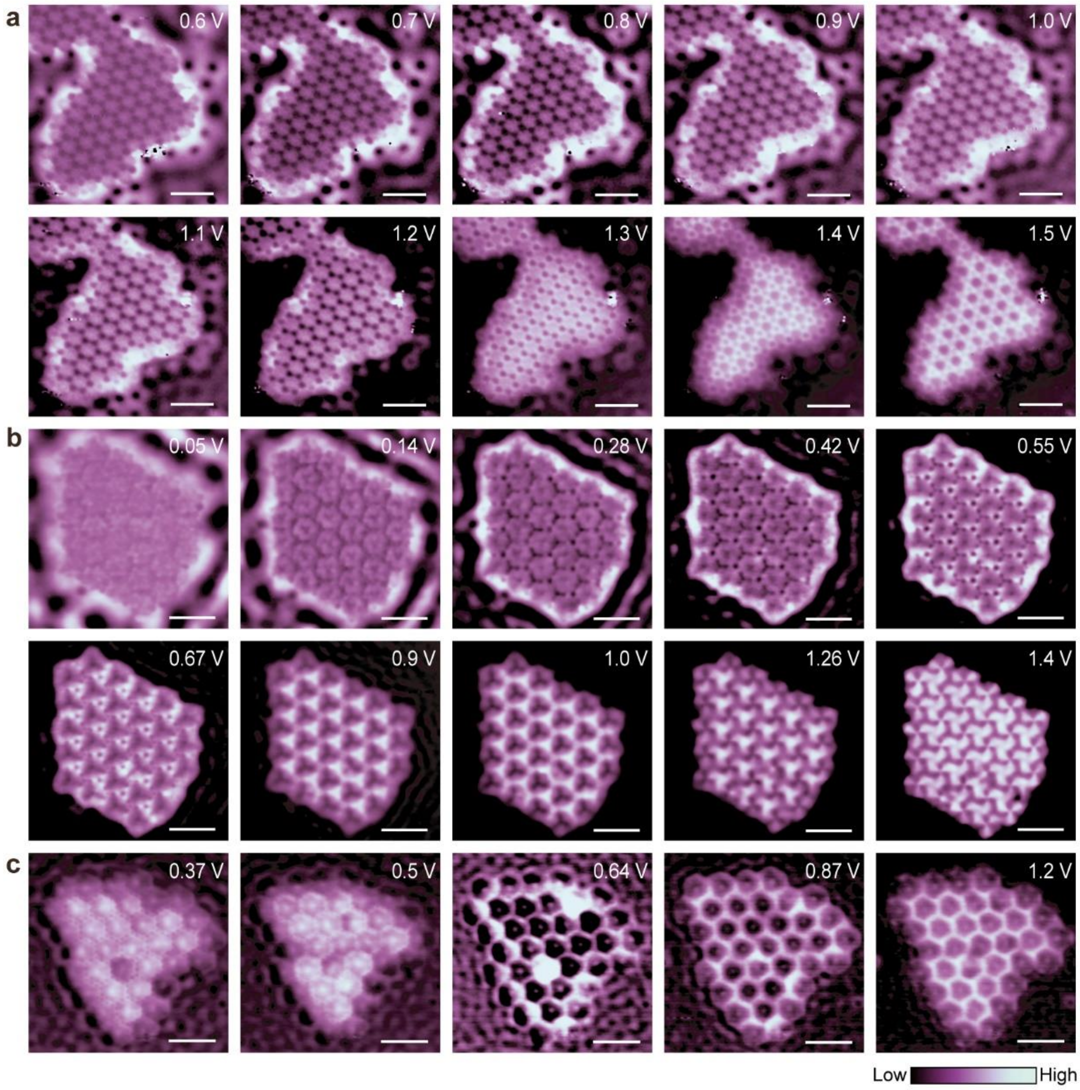

Supplementary Fig. 12 | Detailed d*I*/d*V* maps of the XHOF islands. **a**, The benzene/Br/Ag(111) superlattice, **b**, the M-C66/Br/Ag(111) superlattice, and **c**, the C66/Br/Au(111) superlattice. The mapping bias is marked in each figure. d*I*/d*V* measurement parameters: **a**, I_t = 500 pA; **b**, I_t = 50 pA; **c**, I_t = 10 pA. Scale bars: **a**, 2 nm; **b**, 4 nm; **c**, 4 nm.

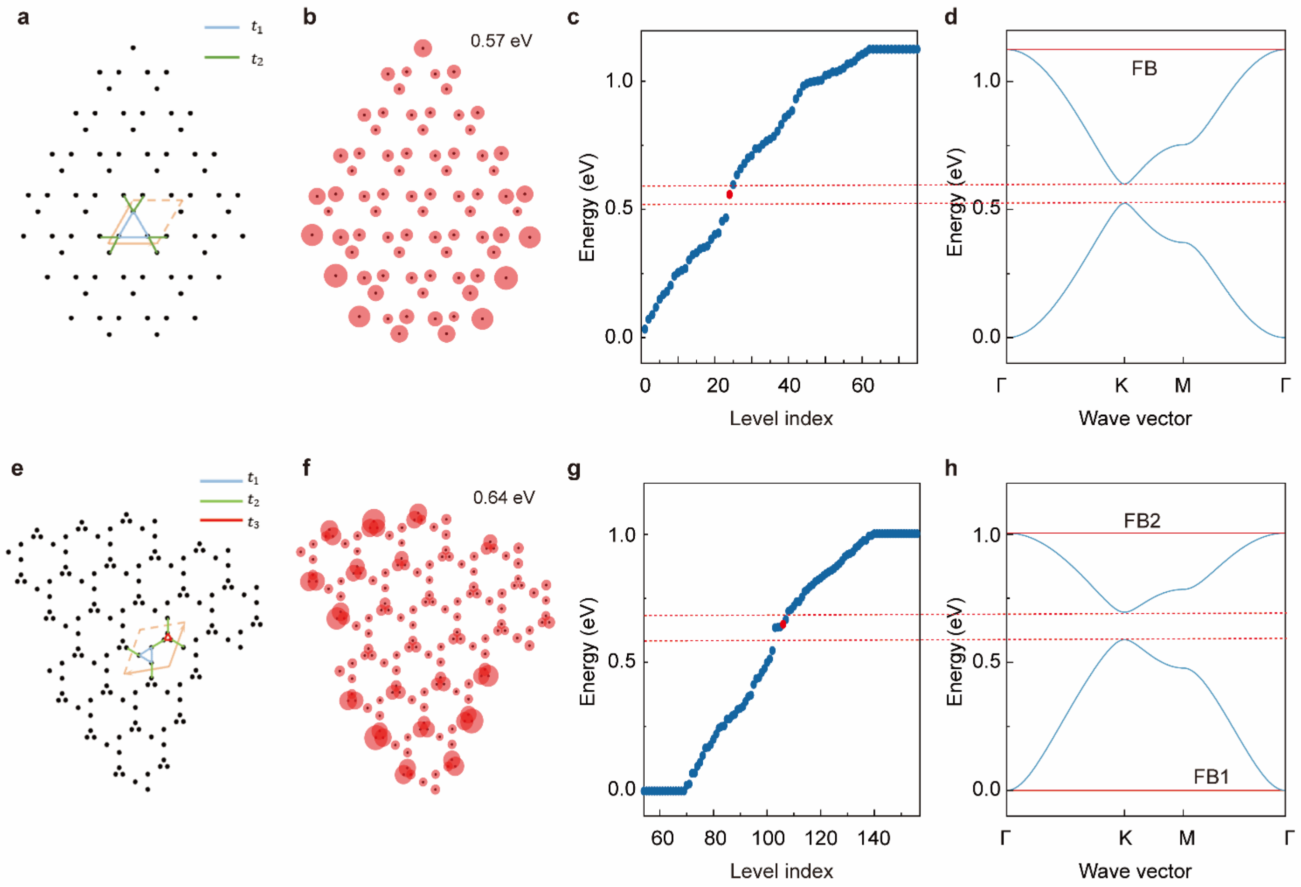

Supplementary Fig. 13 | TB calculations. a, Cluster model of the breathing Kagome lattice, determined experimentally. The unit cell is indicated by the orange parallelogram. The hopping parameter $t_1(t_2)$ is represented by the blue (green) bond. **b**, Simulated LDOS maps of the edge states, same as Fig. 6g in the main text, based on the cluster model of **a** at an energy of 0.57 eV, as indicated by a red dot in **c**. **c**, Calculated energy levels based on the cluster model of **a**. **d**, The *k*-space dispersion of the electronic bands of the breathing kagome lattice, same as Fig. 3d in the main text. **e**, Cluster model of the chiral breathing diatomic-kagome lattice, determined experimentally. The unit cell is enclosed by the orange parallelogram. Hopping parameters $t_1, t_2,$ and t_3 are represented by the blue, green and red bonds, respectively. **f**, Simulated LDOS maps of the edge states, same as Fig. 6k in the main text, based on the cluster model of **a** at an energy of 0.64 eV, as indicated by a red dot in **g**. **g**, Calculated energy levels based on the cluster model of **e**. **h**, The *k*-space dispersion of the electronic bands of the breathing kagome lattice, same as Fig. 3f in the main text. Edge states are emerging in the gap regions for both cases, indicating their nontrivial topology.
