# Supplementary figures and images for: Artificial kagome lattices of Shockley surface states patterned by halogen hydrogen-bonded organic frameworks
Source: Nat Commun. 2024 Apr 6;15:2969. doi: 10.1038/s41467-024-47367-5 (PMC10998891; doi:10.1038/s41467-024-47367-5)

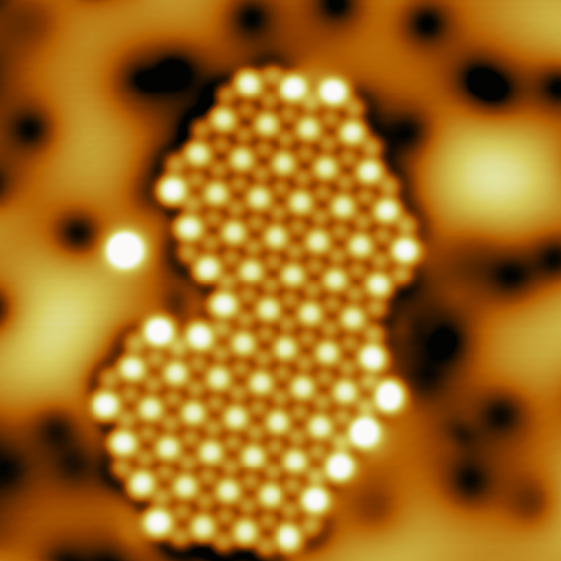

Supplement: Supplementary file 3 — Source data [file 41467_2024_47367_MOESM3_ESM.zip › Fig.2/Fig.2b/20210723-Ag18-4-BrPn-Ag(111)-LHe033 Image Z.bmp]

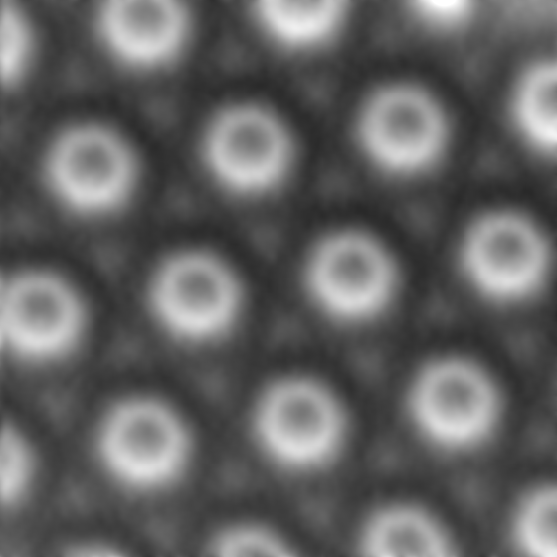

Supplement: Supplementary file 3 — Source data [file 41467_2024_47367_MOESM3_ESM.zip › Fig.2/Fig.2c/20210729-AFM13-4-BrPn-Ag(111)-LHe024 Image.bmp]

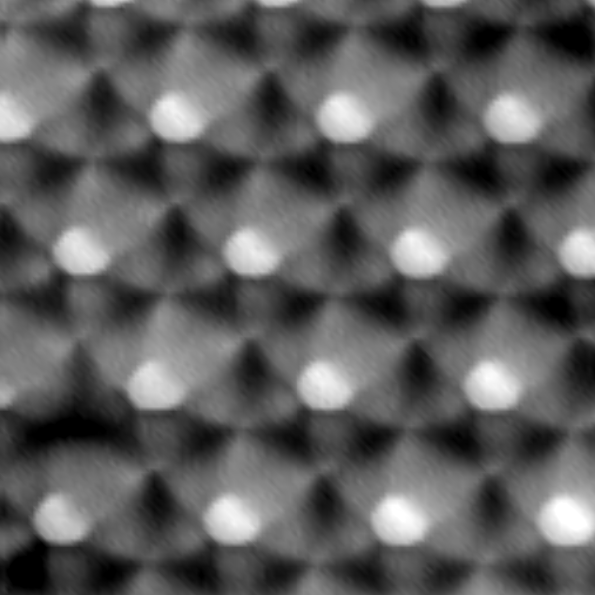

Supplement: Supplementary file 3 — Source data [file 41467_2024_47367_MOESM3_ESM.zip › Fig.2/Fig.2d/20210726 AFM13-4-BrPn-Ag(111)-LHe039 Image OC_M1_Freq._Shift.bmp]

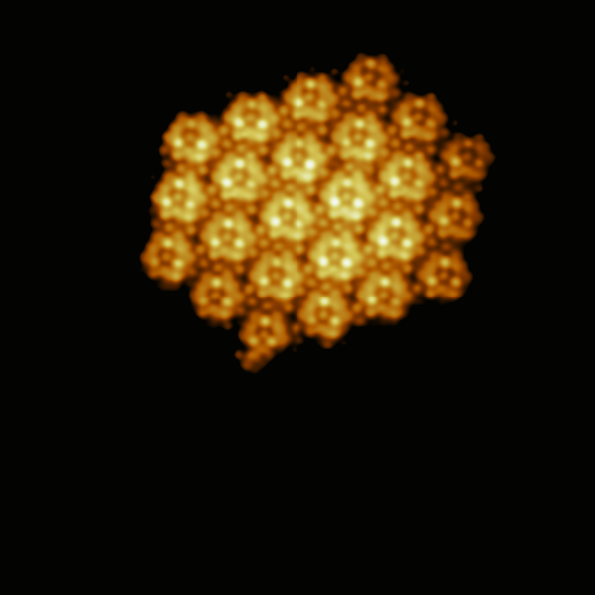

Supplement: Supplementary file 3 — Source data [file 41467_2024_47367_MOESM3_ESM.zip › Fig.2/Fig.2g/202100706_DBP@Ag(111)_LHe_012 Image Z.bmp]

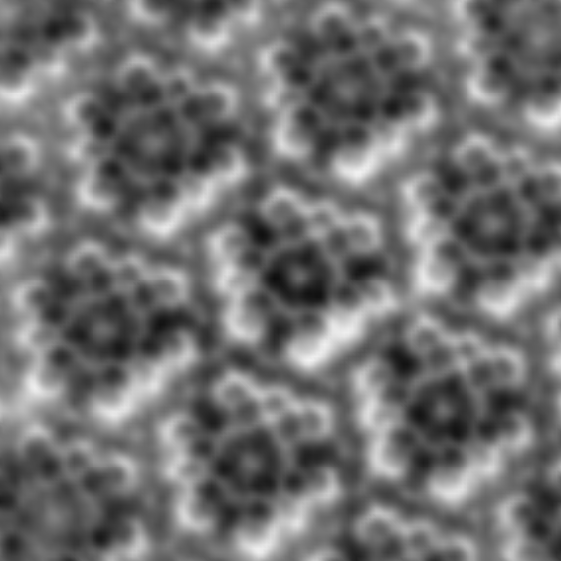

Supplement: Supplementary file 3 — Source data [file 41467_2024_47367_MOESM3_ESM.zip › Fig.2/Fig.2h/202100702_DBP@Ag(111)_LHe_049 Image OC_M1_Freq._Shift (Hz).bmp]

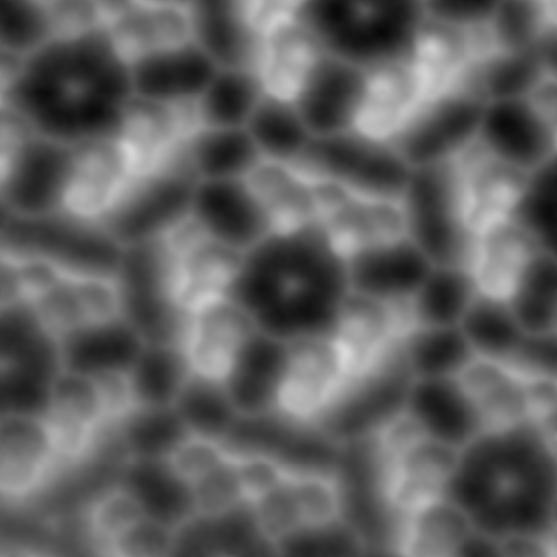

Supplement: Supplementary file 3 — Source data [file 41467_2024_47367_MOESM3_ESM.zip › Fig.2/Fig.2i/202100702_DBP@Ag(111)_LHe_067 Image OC_M1_Freq._Shift.bmp]

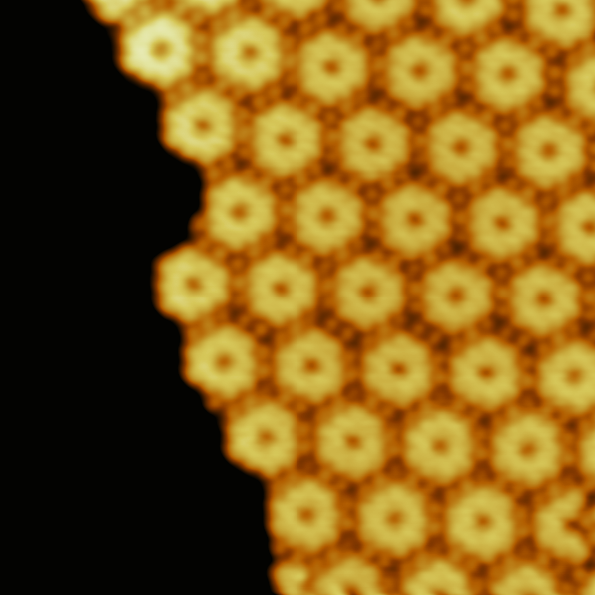

Supplement: Supplementary file 3 — Source data [file 41467_2024_47367_MOESM3_ESM.zip › Fig.2/Fig.2l/20210927_DBP@Au(111)_LHe_046 Image Z (m).bmp]

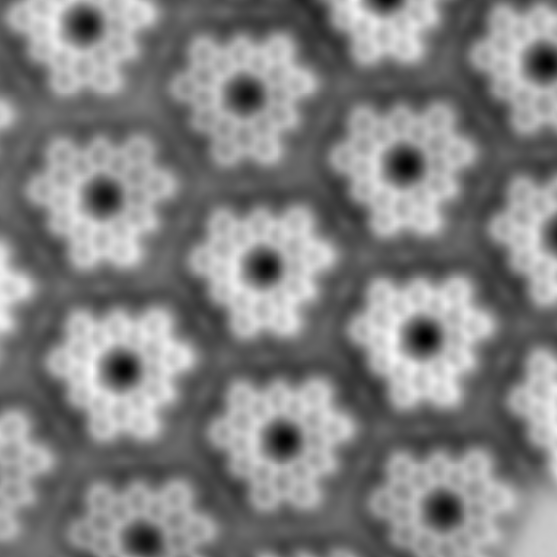

Supplement: Supplementary file 3 — Source data [file 41467_2024_47367_MOESM3_ESM.zip › Fig.2/Fig.2m/20211003_DBP@Au(111)_LHe_080 Image OC_M1_Freq._Shift.bmp]

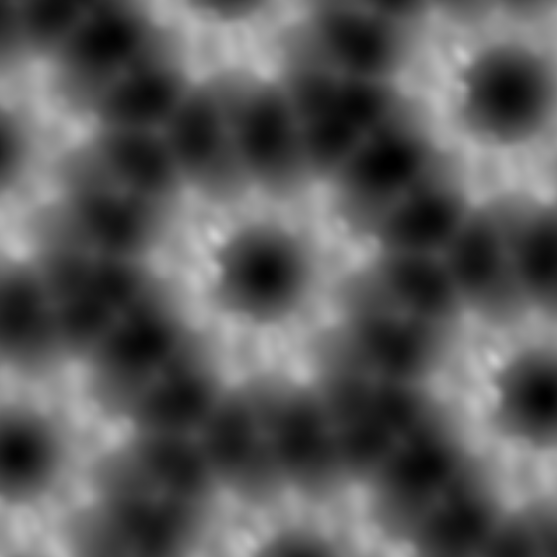

Supplement: Supplementary file 3 — Source data [file 41467_2024_47367_MOESM3_ESM.zip › Fig.2/Fig.2n/20211003_DBP@Au(111)_LHe_086 Image OC_M1_Freq._Shift.bmp]

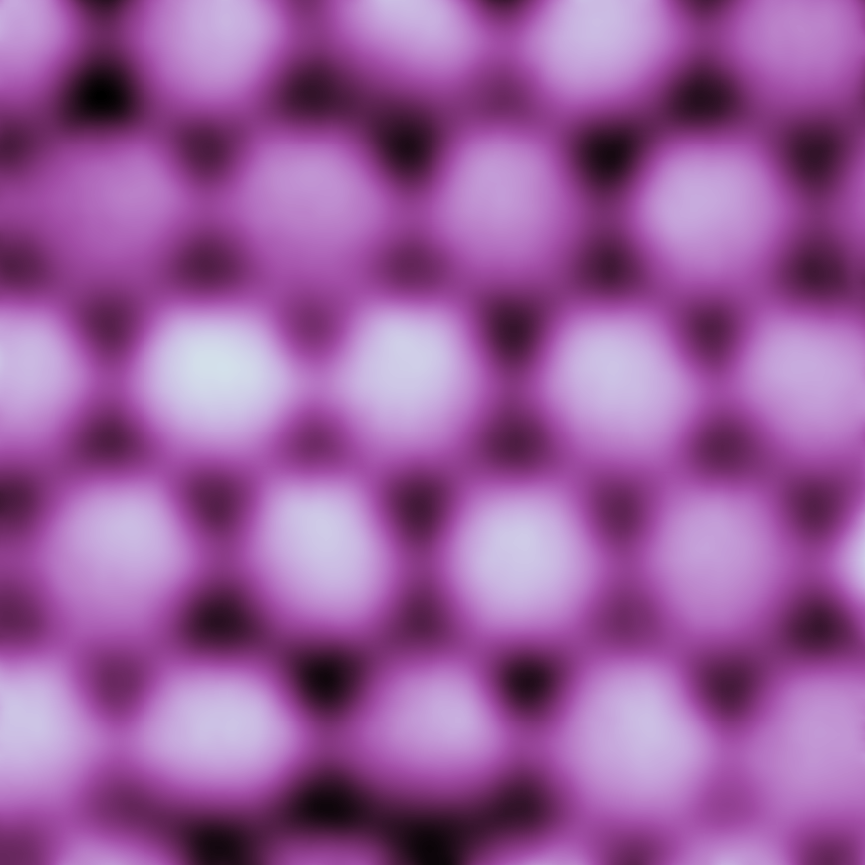

Supplement: Supplementary file 3 — Source data [file 41467_2024_47367_MOESM3_ESM.zip › Fig.4/Fig.4b/202301223_LHe_Agú¿111ú⌐_4BrPn_0149 Image Input_7 (V).stp202301223_LHe_Agú¿111ú⌐_4BrPn_0149 Image Input_7 (V).bmp]

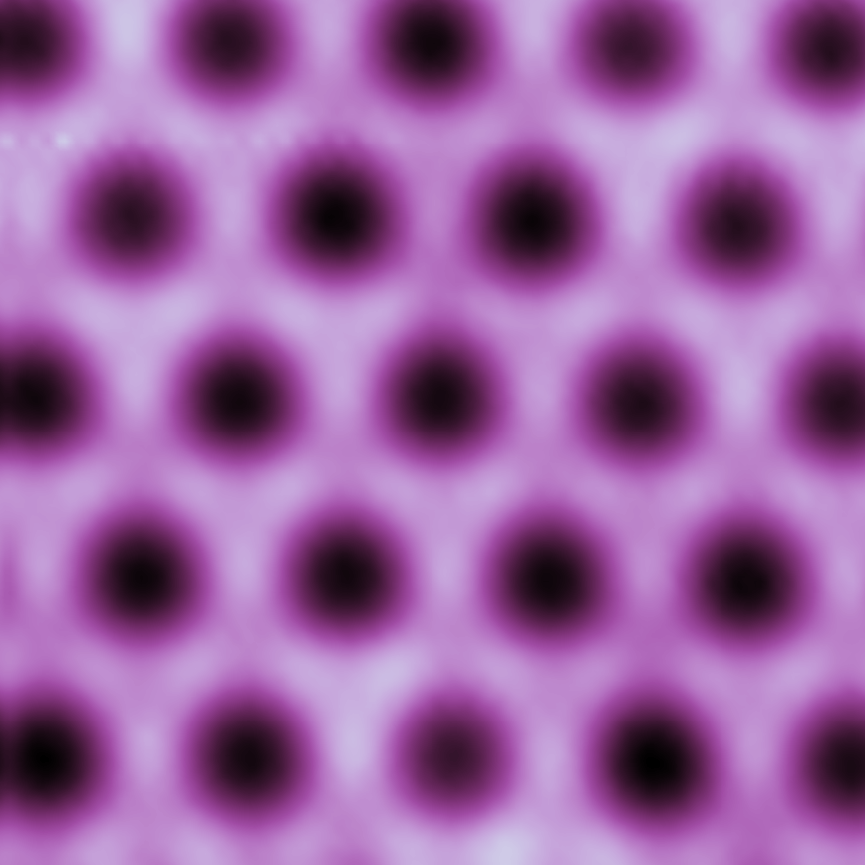

Supplement: Supplementary file 3 — Source data [file 41467_2024_47367_MOESM3_ESM.zip › Fig.4/Fig.4c/202301223_LHe_Agú¿111ú⌐_4BrPn_0144 Image Input_7 (V).stp202301223_LHe_Agú¿111ú⌐_4BrPn_0144 Image Input_7 (V).bmp]

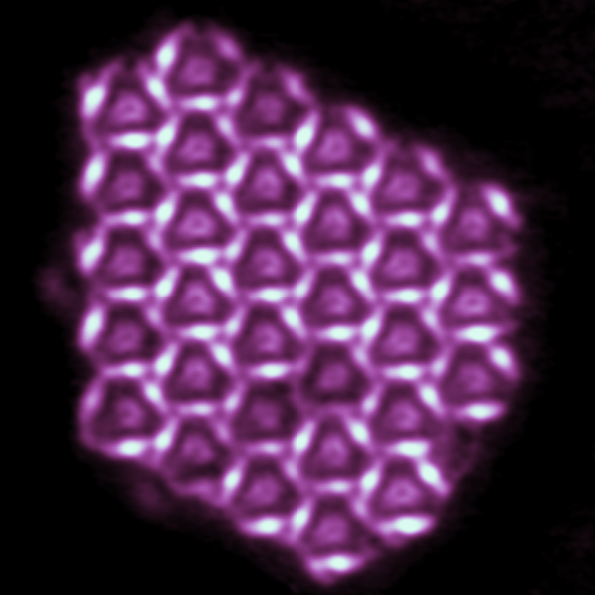

Supplement: Supplementary file 3 — Source data [file 41467_2024_47367_MOESM3_ESM.zip › Fig.4/Fig.4e/202100627_DBP@Ag(111)_LHe_064 Image Input_7 (V)1.bmp]

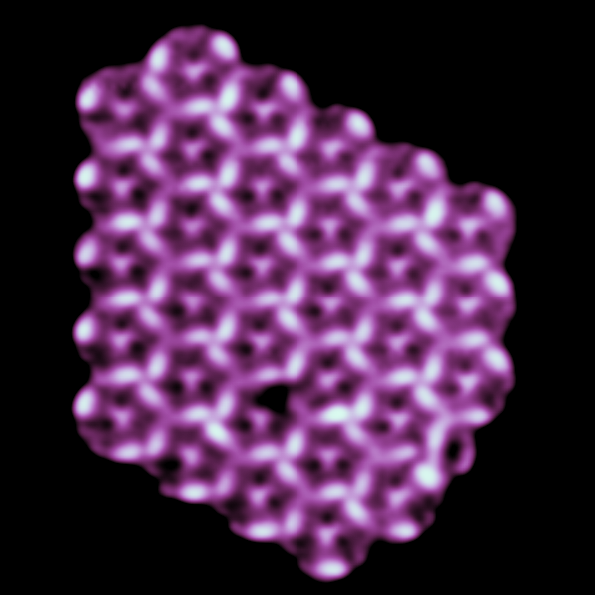

Supplement: Supplementary file 3 — Source data [file 41467_2024_47367_MOESM3_ESM.zip › Fig.4/Fig.4f/202100627_DBP@Ag(111)_LHe_031 Image Input_7 (V)1.bmp]

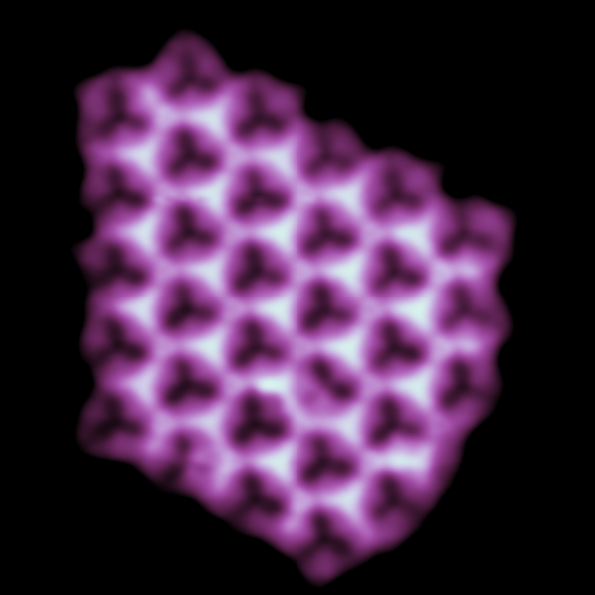

Supplement: Supplementary file 3 — Source data [file 41467_2024_47367_MOESM3_ESM.zip › Fig.4/Fig.4g/202100627_DBP@Ag(111)_LHe_054 Image Input_7 (V)1.bmp]

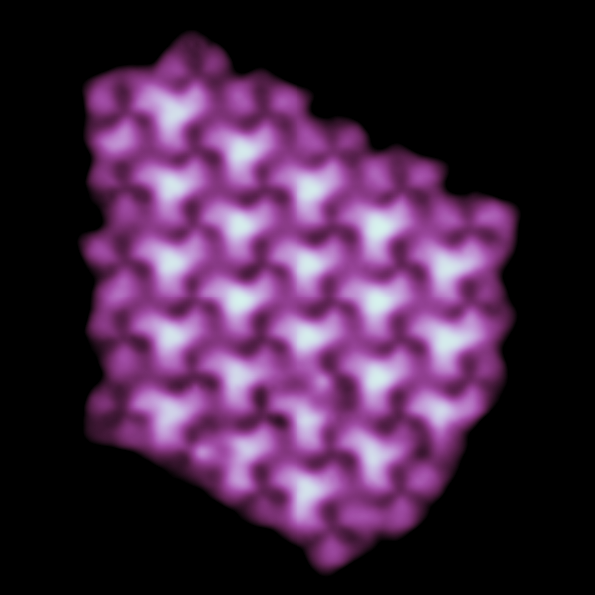

Supplement: Supplementary file 3 — Source data [file 41467_2024_47367_MOESM3_ESM.zip › Fig.4/Fig.4h/202100627_DBP@Ag(111)_LHe_028 Image Input_7 (V)1.bmp]

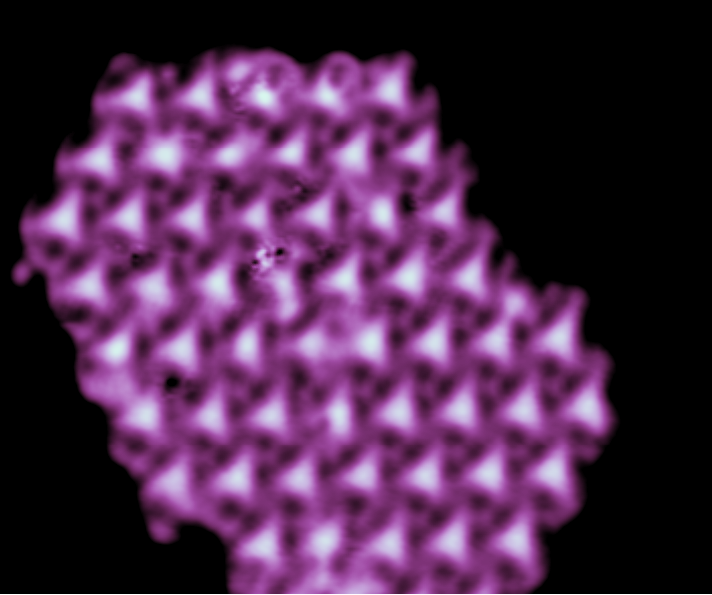

Supplement: Supplementary file 3 — Source data [file 41467_2024_47367_MOESM3_ESM.zip › Fig.4/Fig.4i/202100724_DBP@Ag(111)_LHe_065 Image Input_7 (V)1.bmp]

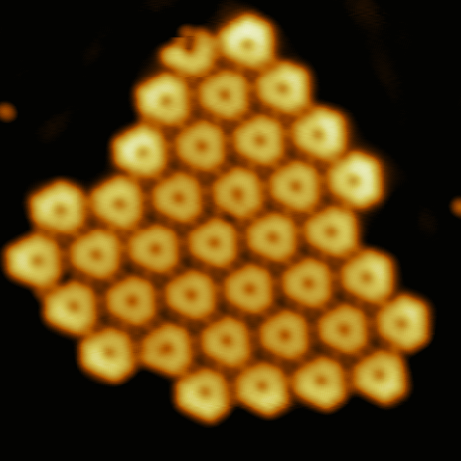

Supplement: Supplementary file 3 — Source data [file 41467_2024_47367_MOESM3_ESM.zip › Fig.5/Fig.5b/20211203_DBP@Au(111)_LHe_035 Image Z (m).stp20211203_DBP@Au(111)_LHe_035 Image Z (m).bmp]

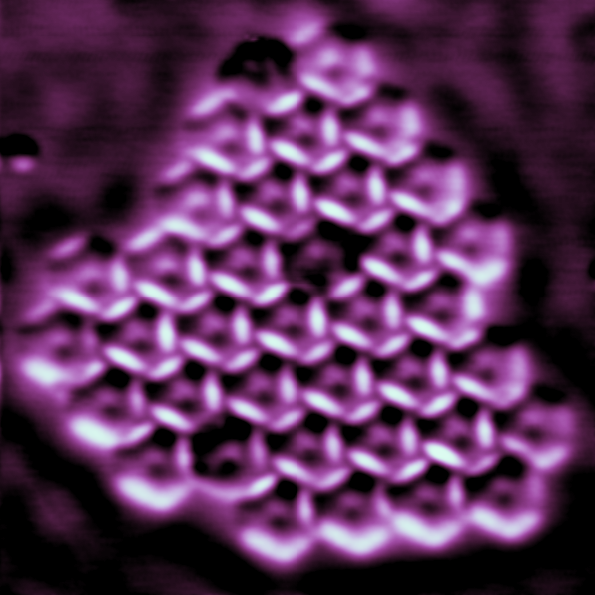

Supplement: Supplementary file 3 — Source data [file 41467_2024_47367_MOESM3_ESM.zip › Fig.5/Fig.5c/20211203_DBP@Au(111)_LHe_023 Image Input_7 (V)1.bmp]

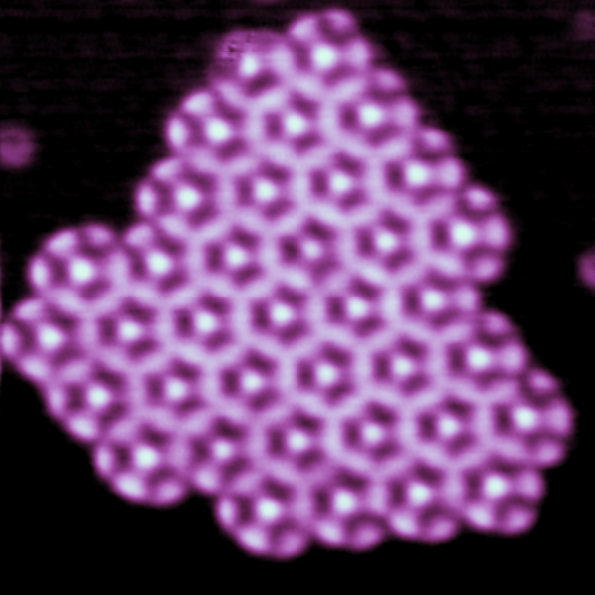

Supplement: Supplementary file 3 — Source data [file 41467_2024_47367_MOESM3_ESM.zip › Fig.5/Fig.5d/20211203_DBP@Au(111)_LHe_027 Image Input_7 (V)1.bmp]

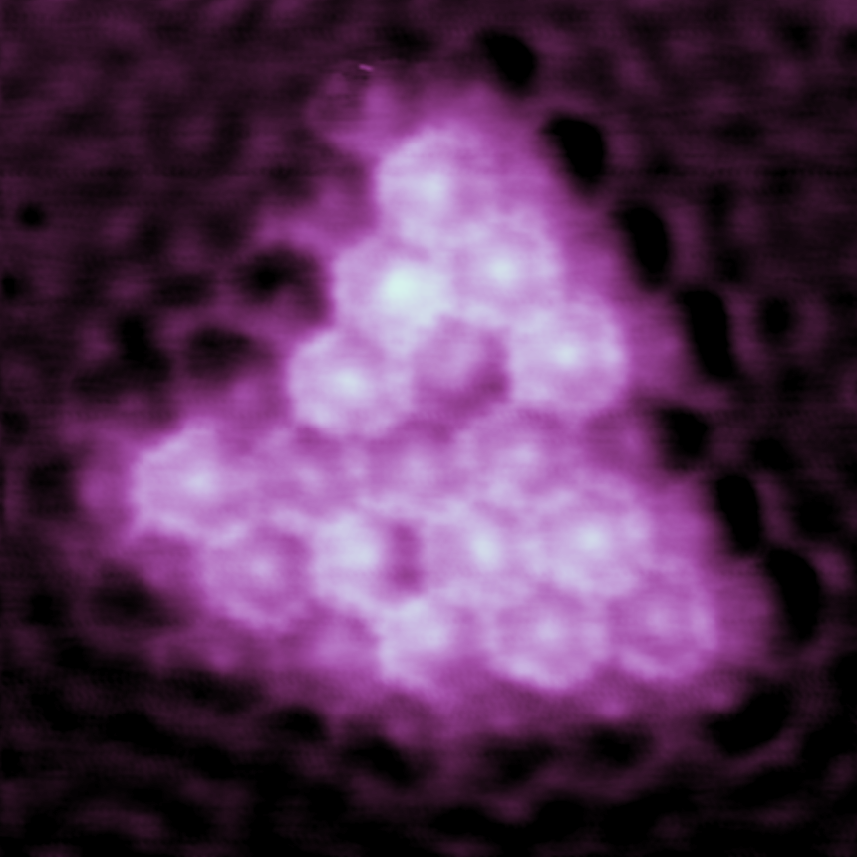

Supplement: Supplementary file 3 — Source data [file 41467_2024_47367_MOESM3_ESM.zip › Fig.5/Fig.5e/20211203_DBP@Au(111)_LHe_024 Image Input_7 (V)0.5V.bmp]

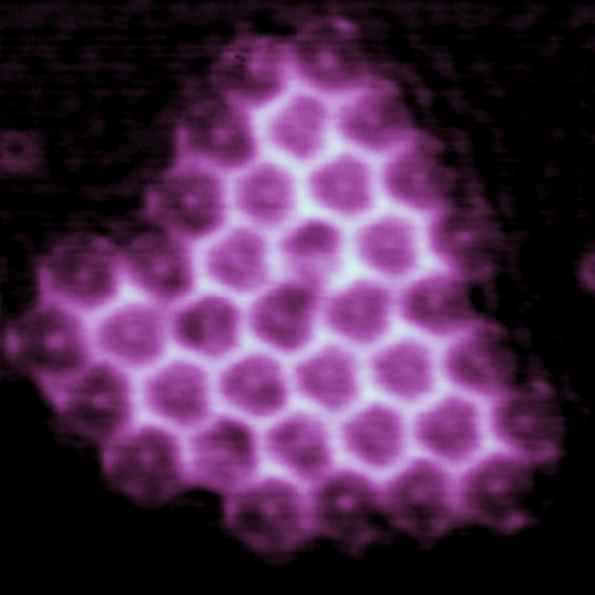

Supplement: Supplementary file 3 — Source data [file 41467_2024_47367_MOESM3_ESM.zip › Fig.5/Fig.5f/20211203_DBP@Au(111)_LHe_026 Image Input_7 (V)1.bmp]

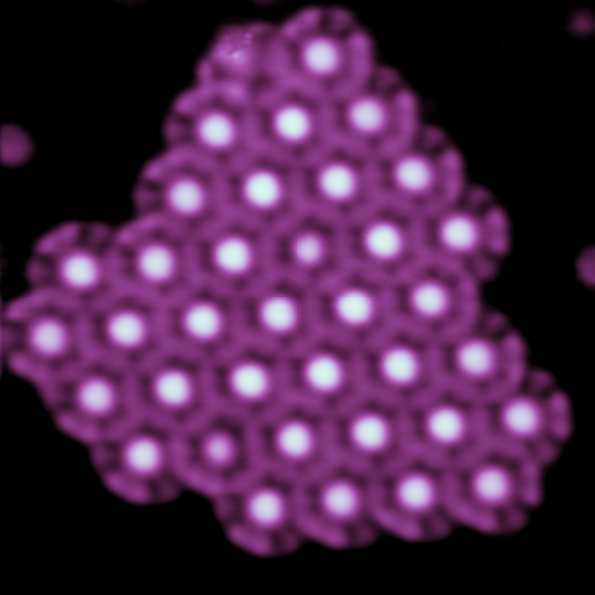

Supplement: Supplementary file 3 — Source data [file 41467_2024_47367_MOESM3_ESM.zip › Fig.5/Fig.5g/20211203_DBP@Au(111)_LHe_029 Image Input_7 (V)3.bmp]

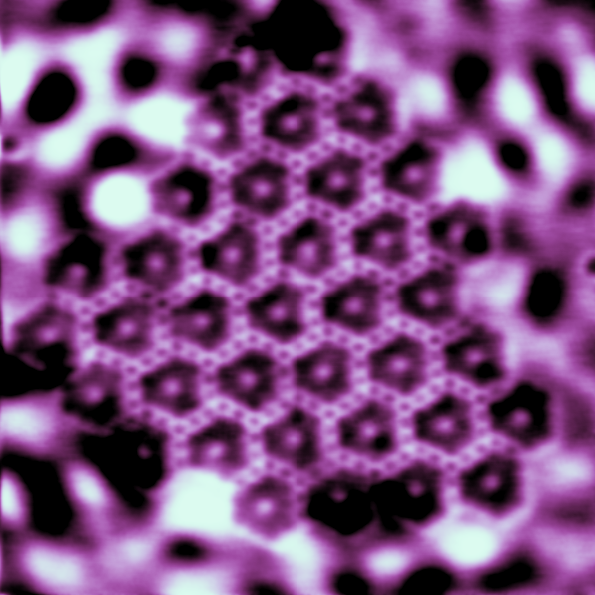

Supplement: Supplementary file 3 — Source data [file 41467_2024_47367_MOESM3_ESM.zip › Fig.5/Fig.5i/20211203_DBP@Au(111)_LHe_035 Image Input_7 (V)1.bmp]

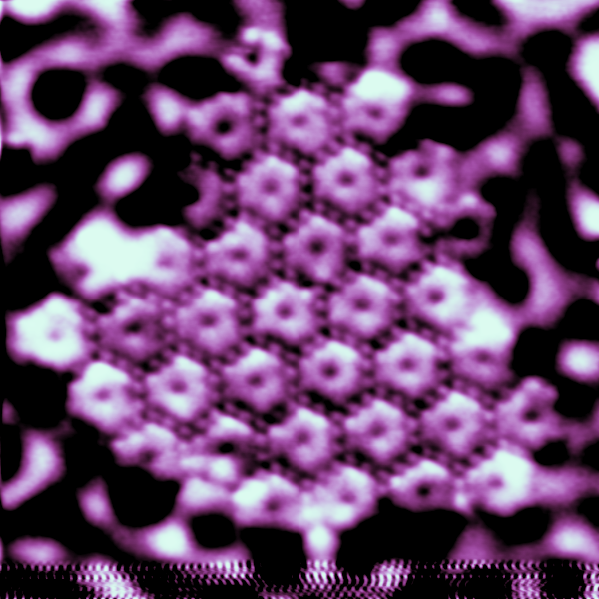

Supplement: Supplementary file 3 — Source data [file 41467_2024_47367_MOESM3_ESM.zip › Fig.5/Fig.5j/20211203_DBP@Au(111)_LHe_036 Image Input_7 (V)1.bmp]

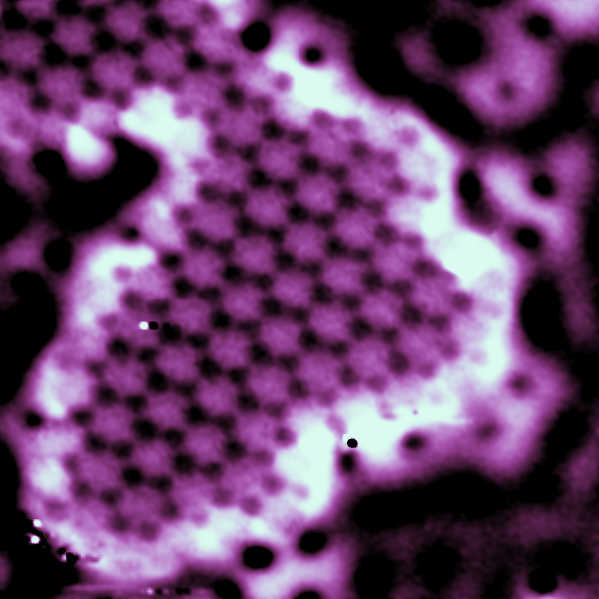

Supplement: Supplementary file 3 — Source data [file 41467_2024_47367_MOESM3_ESM.zip › Fig.6/Fig.6b/AFM13-4-BrPn-Ag(111)-LHe015 Image LI_Demod_1_X (A).bmpAFM13-4-BrPn-Ag(111)-LHe015 Image LI_Demod_1_X (A)1.bmp]

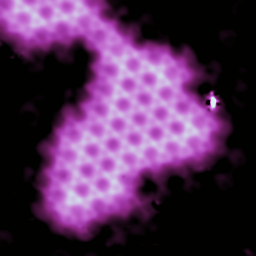

Supplement: Supplementary file 3 — Source data [file 41467_2024_47367_MOESM3_ESM.zip › Fig.6/Fig.6d/AFM13-4-BrPn-Ag(111)-LHe006 Image LI_Demod_1_X (A) 1.5 V.bmp]

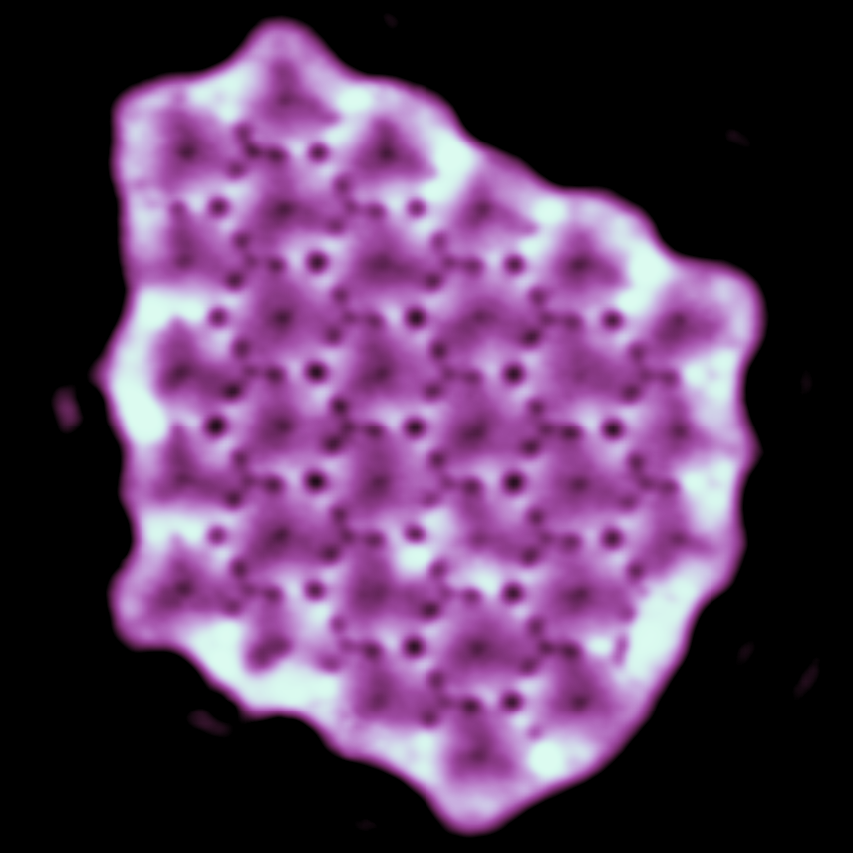

Supplement: Supplementary file 3 — Source data [file 41467_2024_47367_MOESM3_ESM.zip › Fig.6/Fig.6f/202100627_DBP@Ag(111)_LHe_062 Image Input_7 (V).bmp]

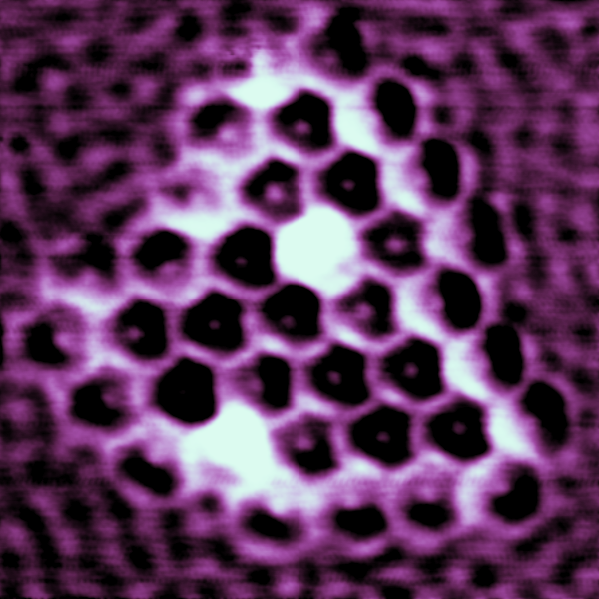

Supplement: Supplementary file 3 — Source data [file 41467_2024_47367_MOESM3_ESM.zip › Fig.6/Fig.6j/20211203_DBP@Au(111)_LHe_025 Image Input_7 (V).bmp20211203_DBP@Au(111)_LHe_025 Image Input_7 (V)1.bmp]

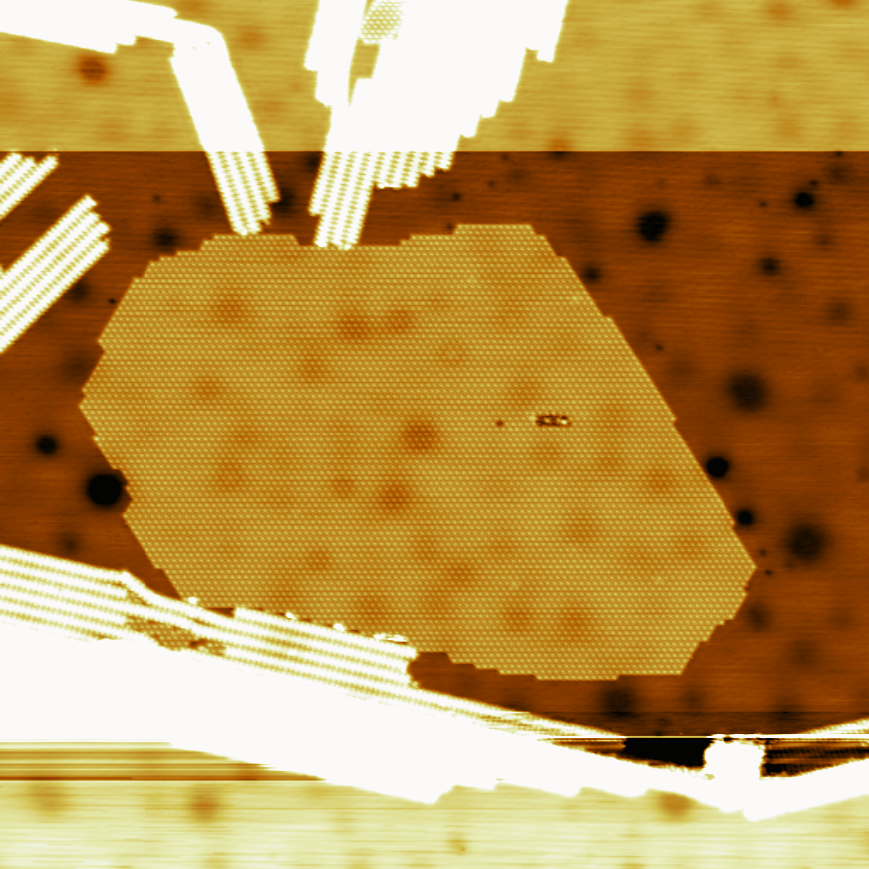

Supplement: Supplementary file 3 — Source data [file 41467_2024_47367_MOESM3_ESM.zip › Supplementary Fig.1/Supplementary Fig.1a/202301120_LHe_Agú¿100ú⌐_4BrPn_0094 Image Z.bmp]

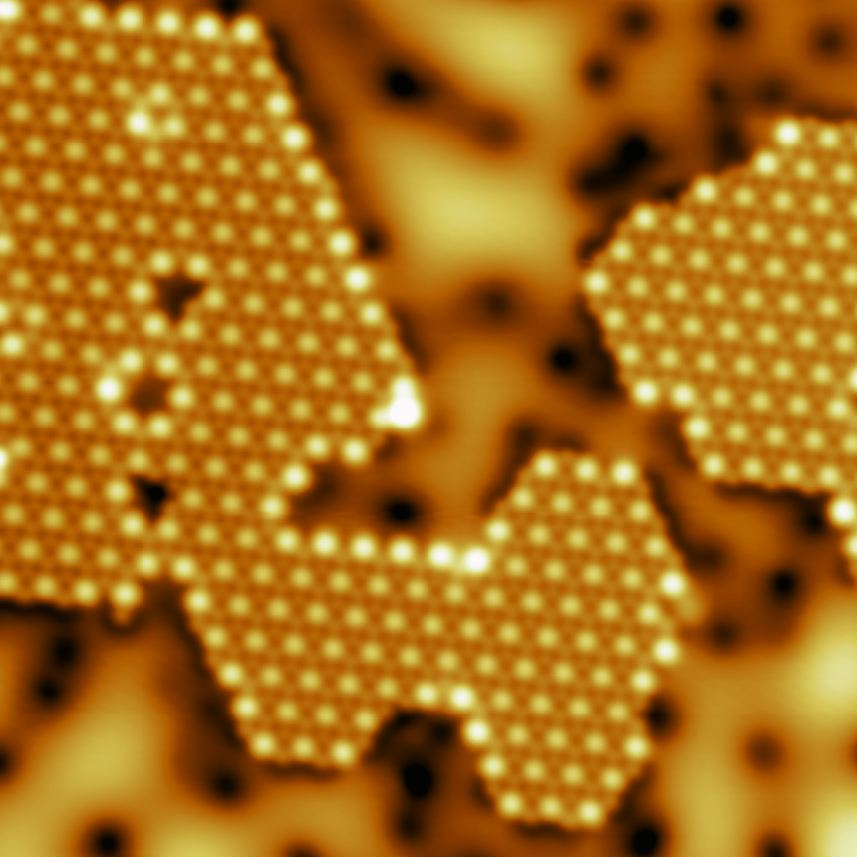

Supplement: Supplementary file 3 — Source data [file 41467_2024_47367_MOESM3_ESM.zip › Supplementary Fig.1/Supplementary Fig.1b/20210723Ag18-4-BrPn-Ag(111)-LHe043 Image Z (m).bmpAg18-4-BrPn-Ag(111)-LHe043 Image Z (m).bmp]

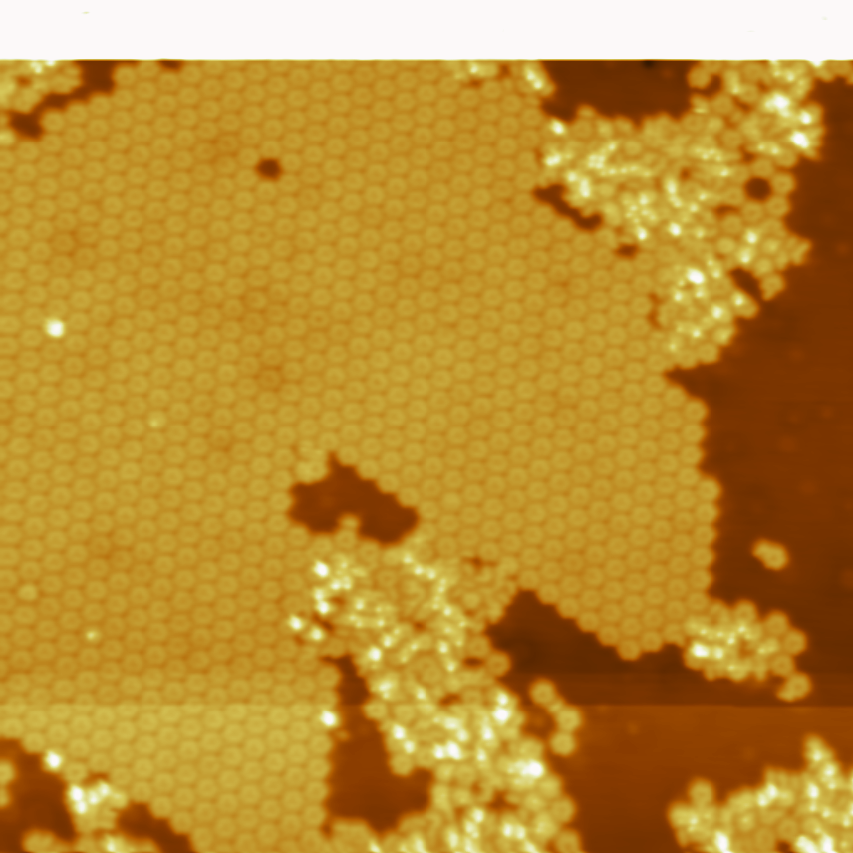

Supplement: Supplementary file 3 — Source data [file 41467_2024_47367_MOESM3_ESM.zip › Supplementary Fig.1/Supplementary Fig.1c/20221107_DBP_Ag(111)_LN2_0019 Image Z (m).bmp]

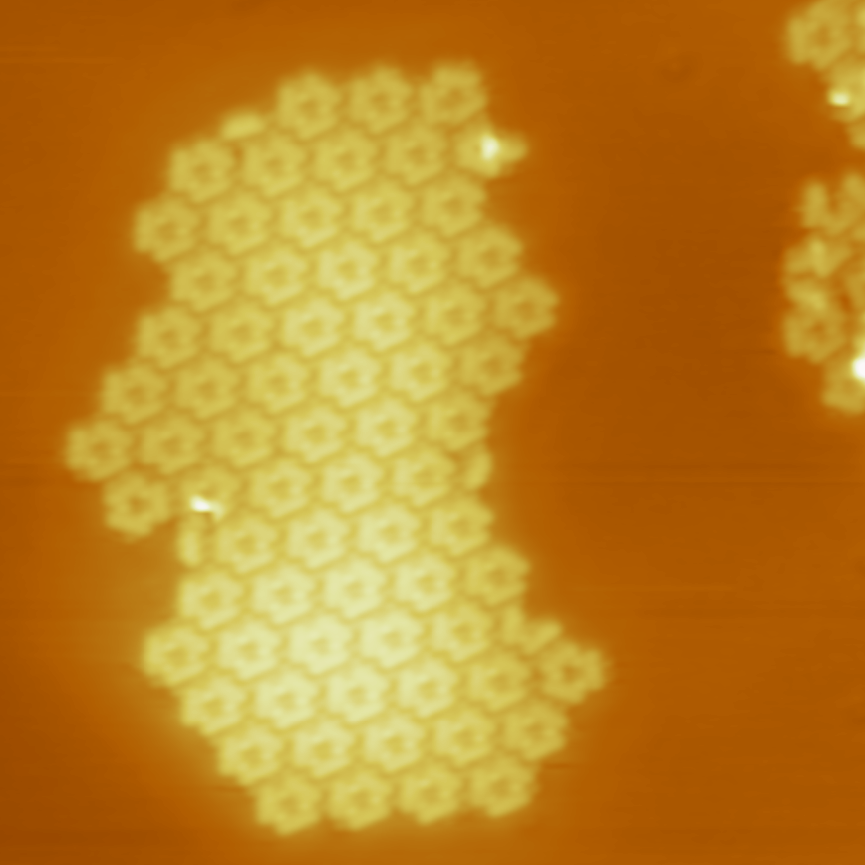

Supplement: Supplementary file 3 — Source data [file 41467_2024_47367_MOESM3_ESM.zip › Supplementary Fig.1/Supplementary Fig.1d/202100713_DBP@Ag(111)_LN2_020 Image Z(m).bmp]

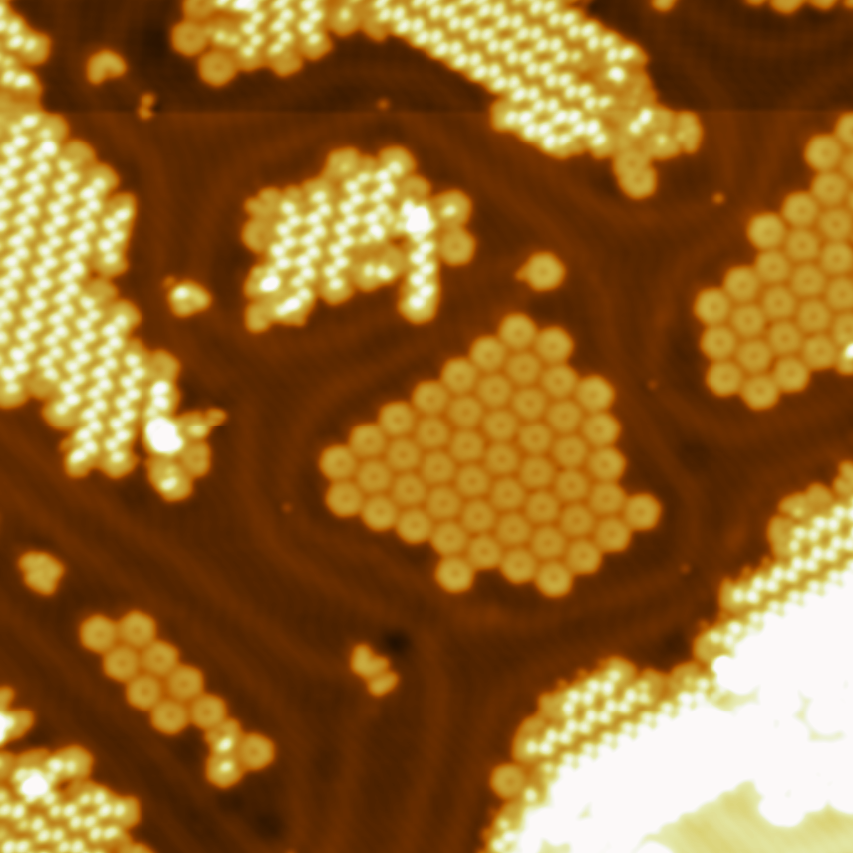

Supplement: Supplementary file 3 — Source data [file 41467_2024_47367_MOESM3_ESM.zip › Supplementary Fig.1/Supplementary Fig.1e/20211130_DBP@Au(111)_LHe_003 Image Z (m).bmp]

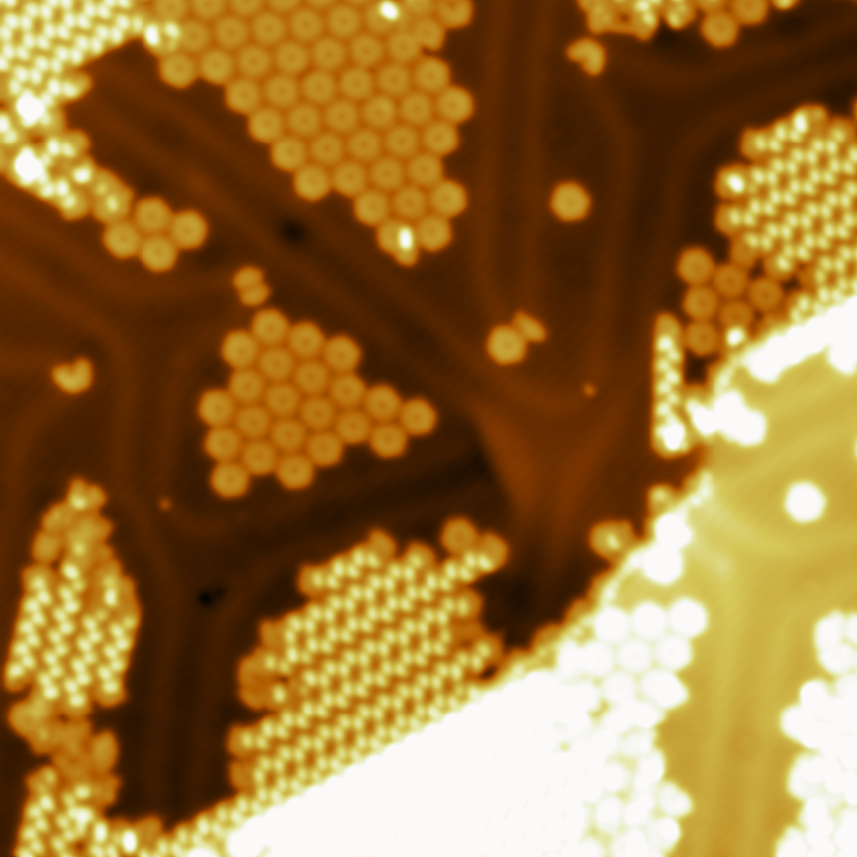

Supplement: Supplementary file 3 — Source data [file 41467_2024_47367_MOESM3_ESM.zip › Supplementary Fig.1/Supplementary Fig.1f/20211203_DBP@Au(111)_LHe_060 Image Z (m).bmp]

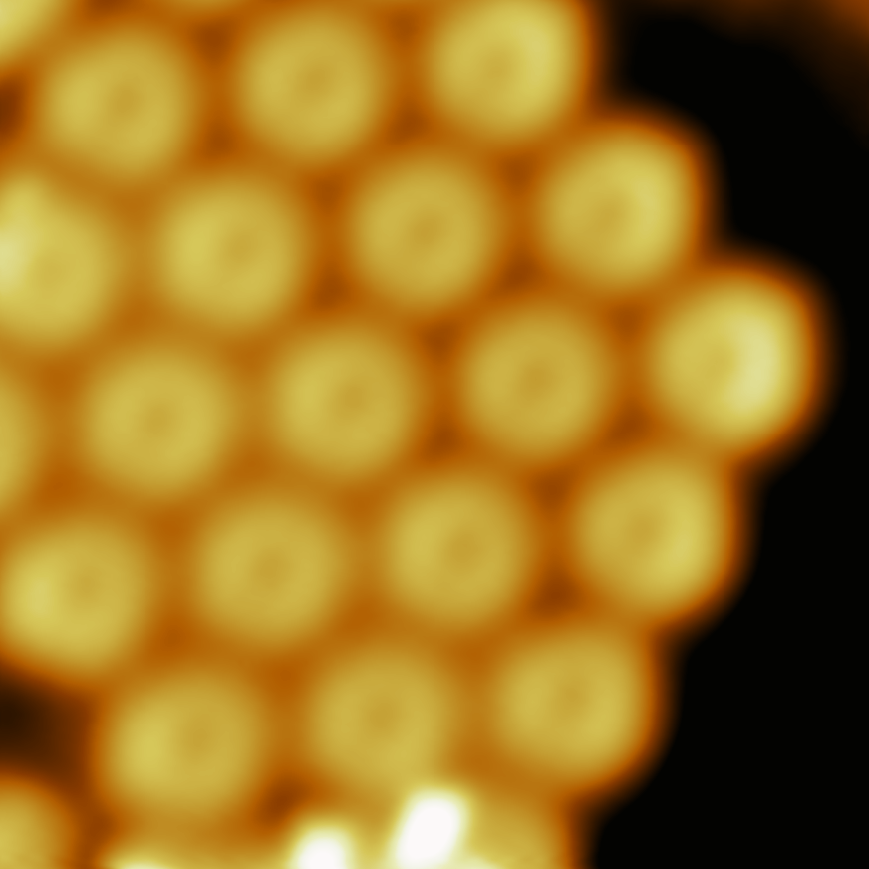

Supplement: Supplementary file 3 — Source data [file 41467_2024_47367_MOESM3_ESM.zip › Supplementary Fig.10/Supplementary Fig.10a/20211125_DBP@Au(111)_LHe_017 Image Z(m).bmp]

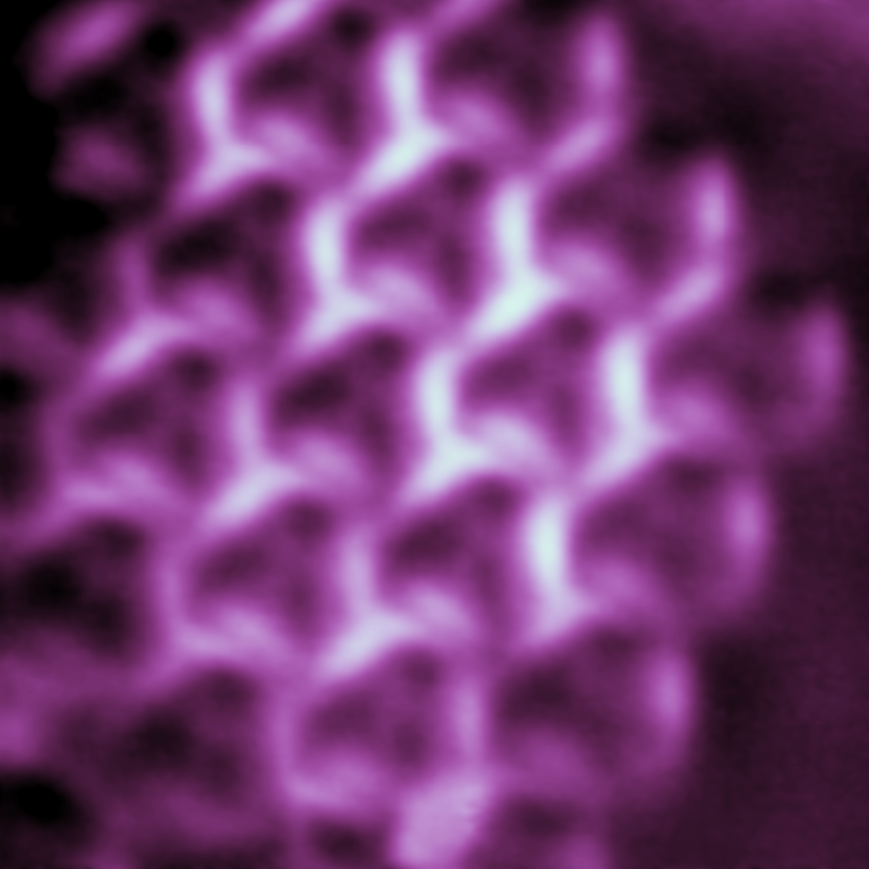

Supplement: Supplementary file 3 — Source data [file 41467_2024_47367_MOESM3_ESM.zip › Supplementary Fig.10/Supplementary Fig.10d/20211125_DBP@Au(111)_LHe_017 Image Input_7.bmp]

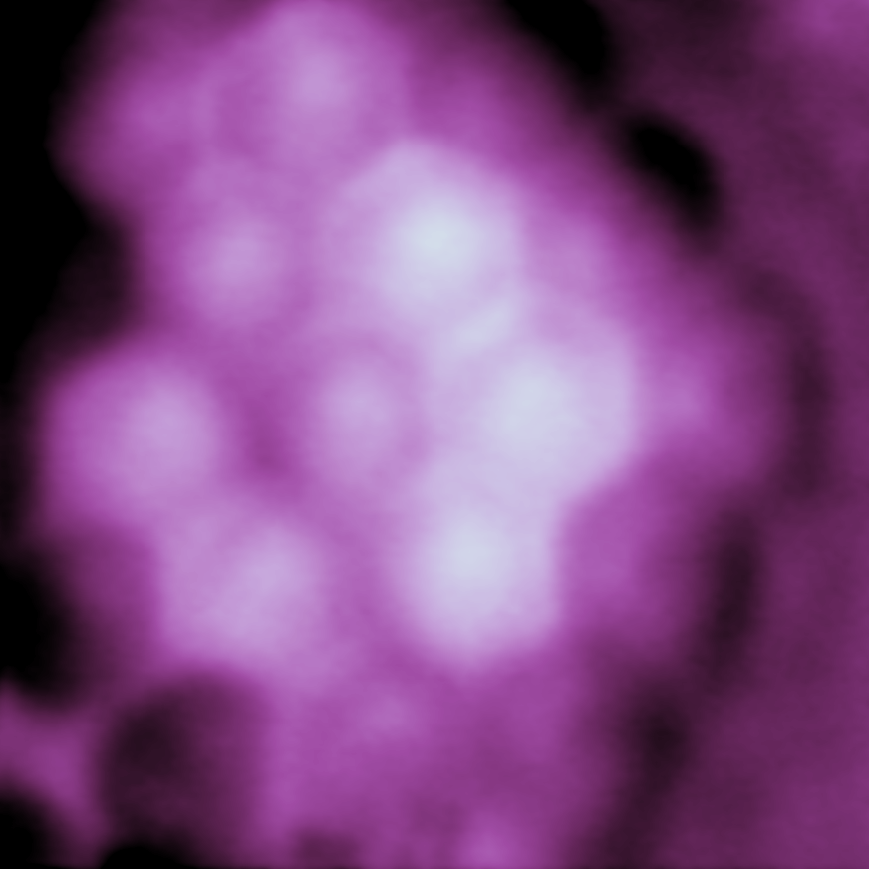

Supplement: Supplementary file 3 — Source data [file 41467_2024_47367_MOESM3_ESM.zip › Supplementary Fig.10/Supplementary Fig.10e/20211125_DBP@Au(111)_LHe_021 Image Input_7.bmp]

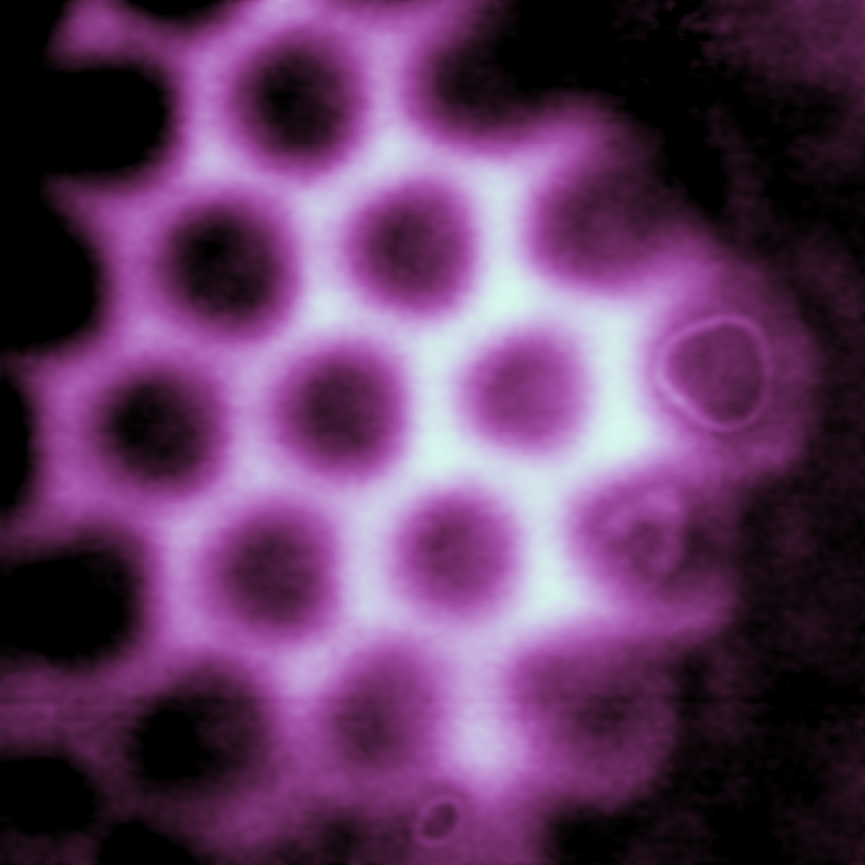

Supplement: Supplementary file 3 — Source data [file 41467_2024_47367_MOESM3_ESM.zip › Supplementary Fig.10/Supplementary Fig.10f/20211125_DBP@Au(111)_LHe_022 Image Input_7.bmp]

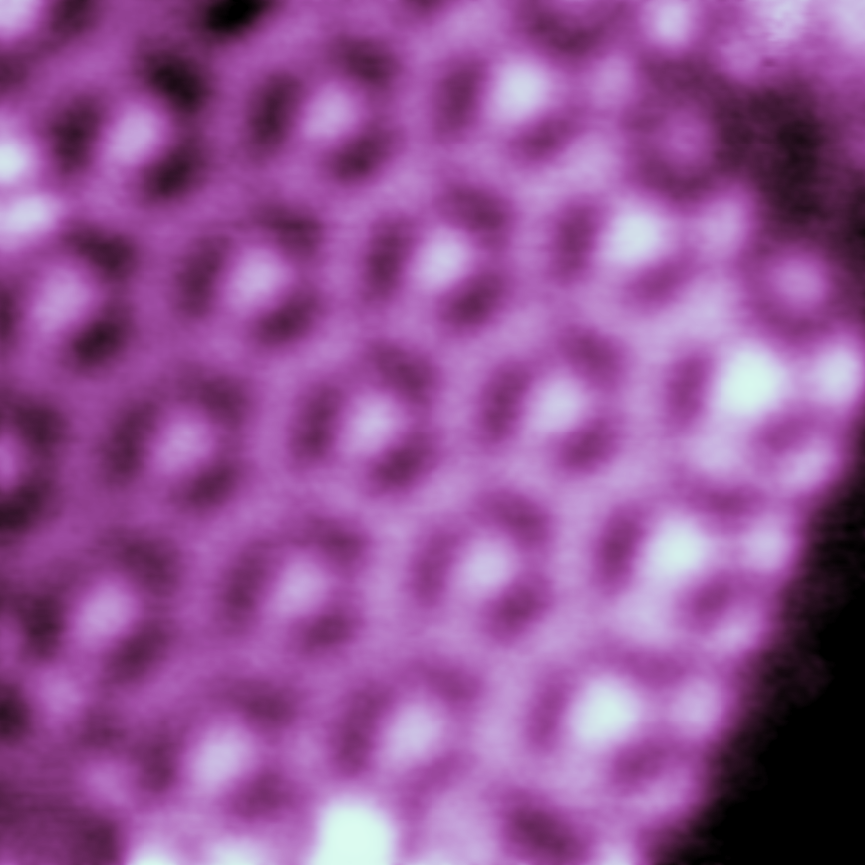

Supplement: Supplementary file 3 — Source data [file 41467_2024_47367_MOESM3_ESM.zip › Supplementary Fig.10/Supplementary Fig.10g/20211125_DBP@Au(111)_LHe_036 Image Input_7.bmp]

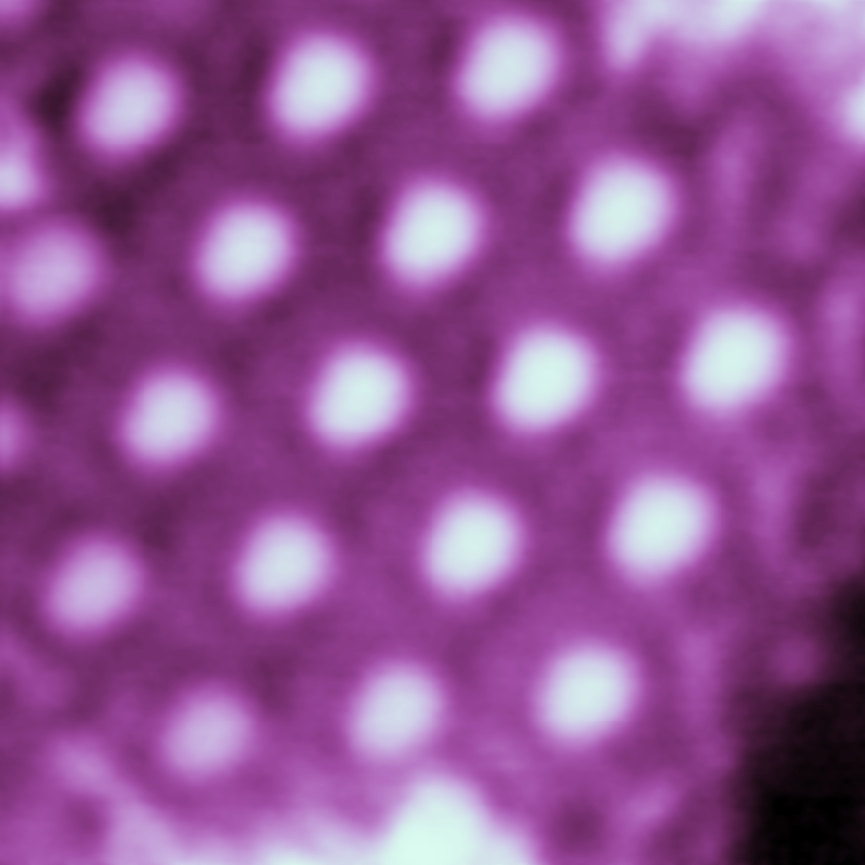

Supplement: Supplementary file 3 — Source data [file 41467_2024_47367_MOESM3_ESM.zip › Supplementary Fig.10/Supplementary Fig.10h/20211125_DBP@Au(111)_LHe_037 Image Input_7.bmp]

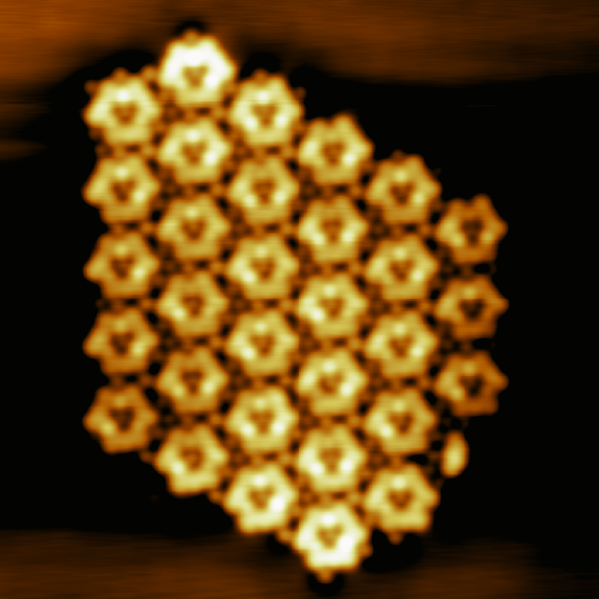

Supplement: Supplementary file 3 — Source data [file 41467_2024_47367_MOESM3_ESM.zip › Supplementary Fig.11/Supplementary Fig.11a/202100627_DBP@Ag(111)_LHe_058 Image1 Z (m).bmp]

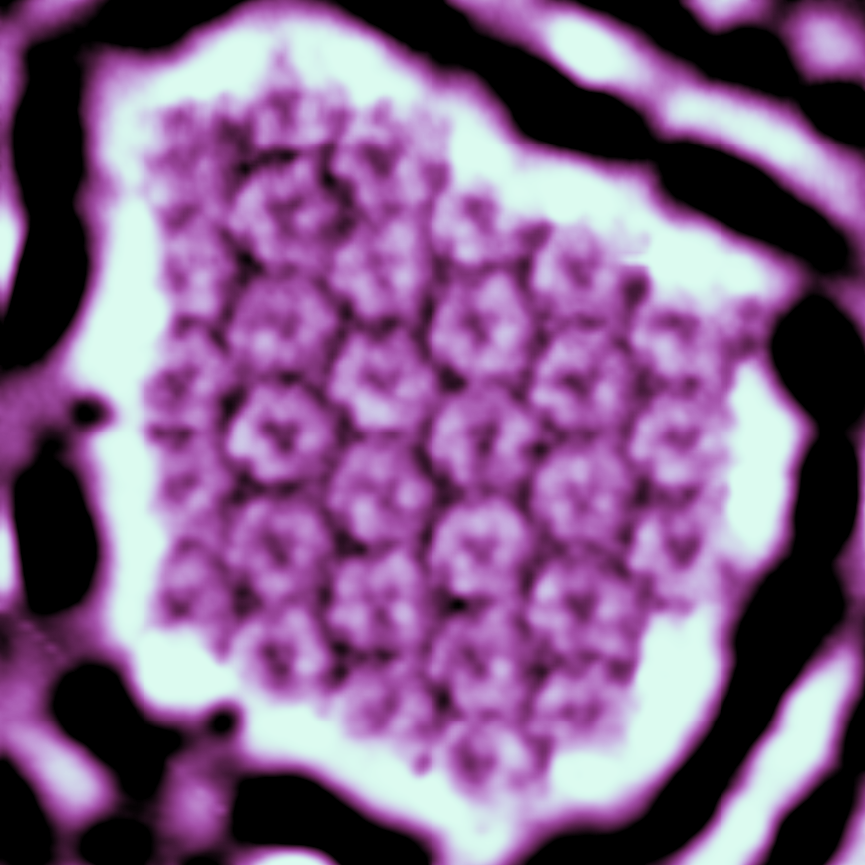

Supplement: Supplementary file 3 — Source data [file 41467_2024_47367_MOESM3_ESM.zip › Supplementary Fig.11/Supplementary Fig.11b/202100627_DBP@Ag(111)_LHe_060 Image Input_7.bmp]

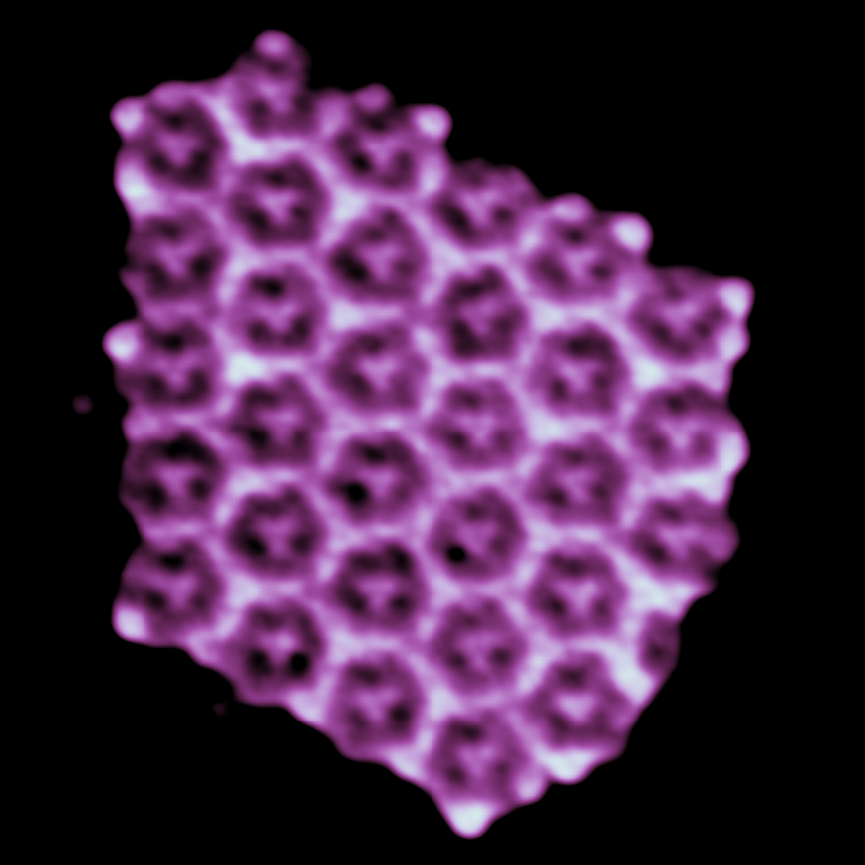

Supplement: Supplementary file 3 — Source data [file 41467_2024_47367_MOESM3_ESM.zip › Supplementary Fig.11/Supplementary Fig.11c/202100627_DBP@Ag(111)_LHe_045 Image Input_7.bmp]

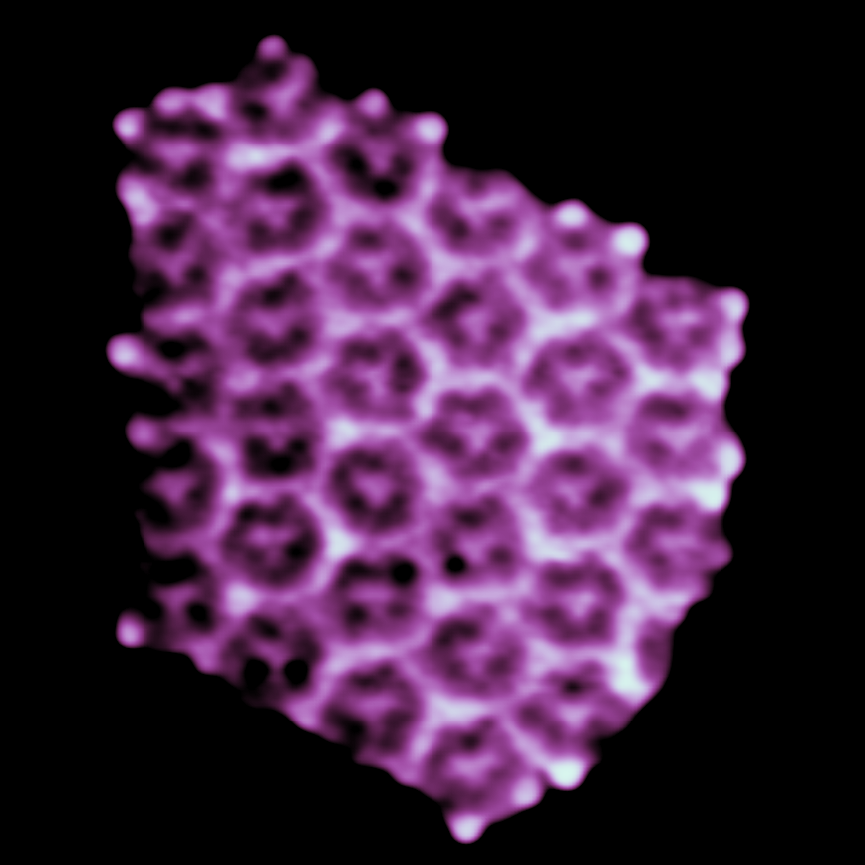

Supplement: Supplementary file 3 — Source data [file 41467_2024_47367_MOESM3_ESM.zip › Supplementary Fig.11/Supplementary Fig.11d/202100627_DBP@Ag(111)_LHe_046 Image Input_7.bmp]

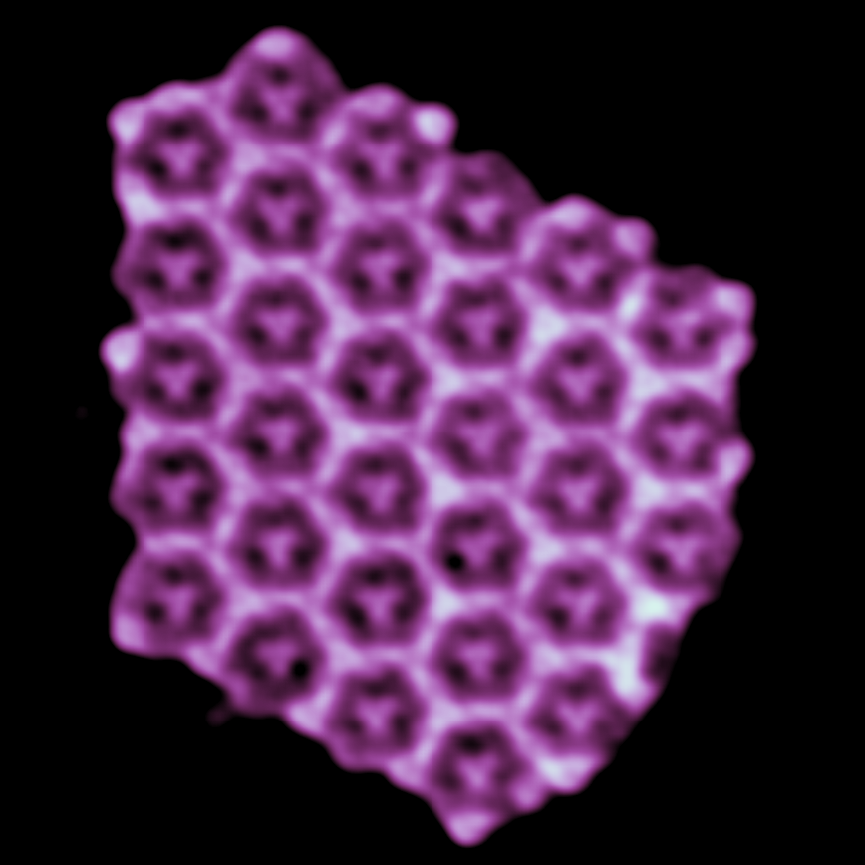

Supplement: Supplementary file 3 — Source data [file 41467_2024_47367_MOESM3_ESM.zip › Supplementary Fig.11/Supplementary Fig.11e/202100627_DBP@Ag(111)_LHe_044 Image Input_7.bmp]

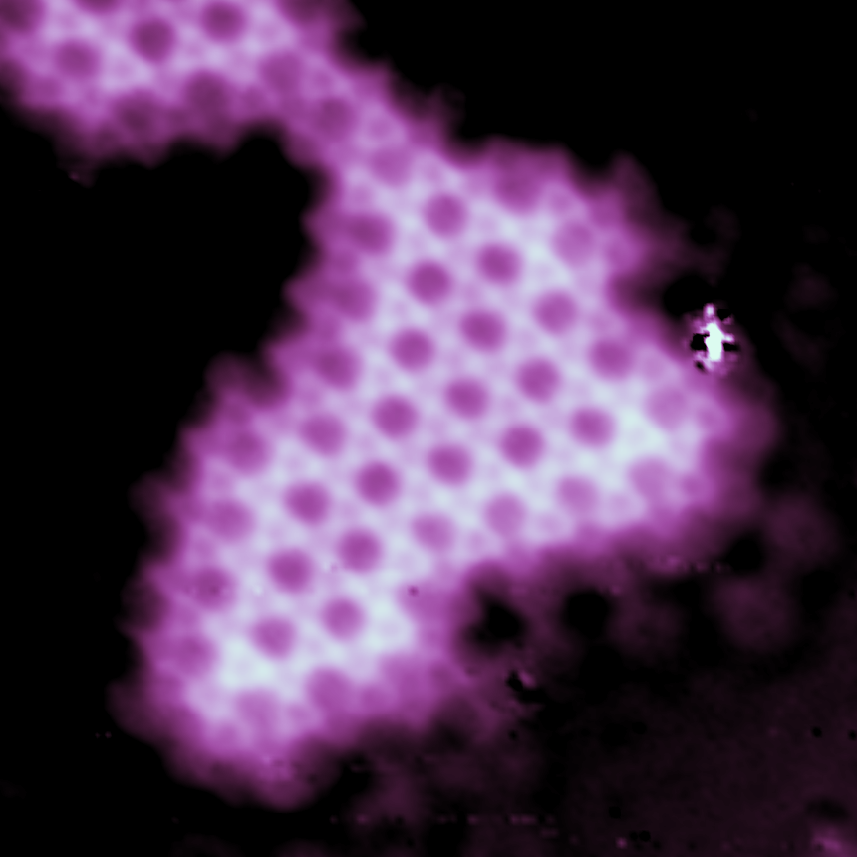

Supplement: Supplementary file 3 — Source data [file 41467_2024_47367_MOESM3_ESM.zip › Supplementary Fig.12/Supplementary Fig.12a/AFM13-4-BrPn-Ag(111)-LHe006 Image LI_Demod_1_X (A)1.5V.bmp]

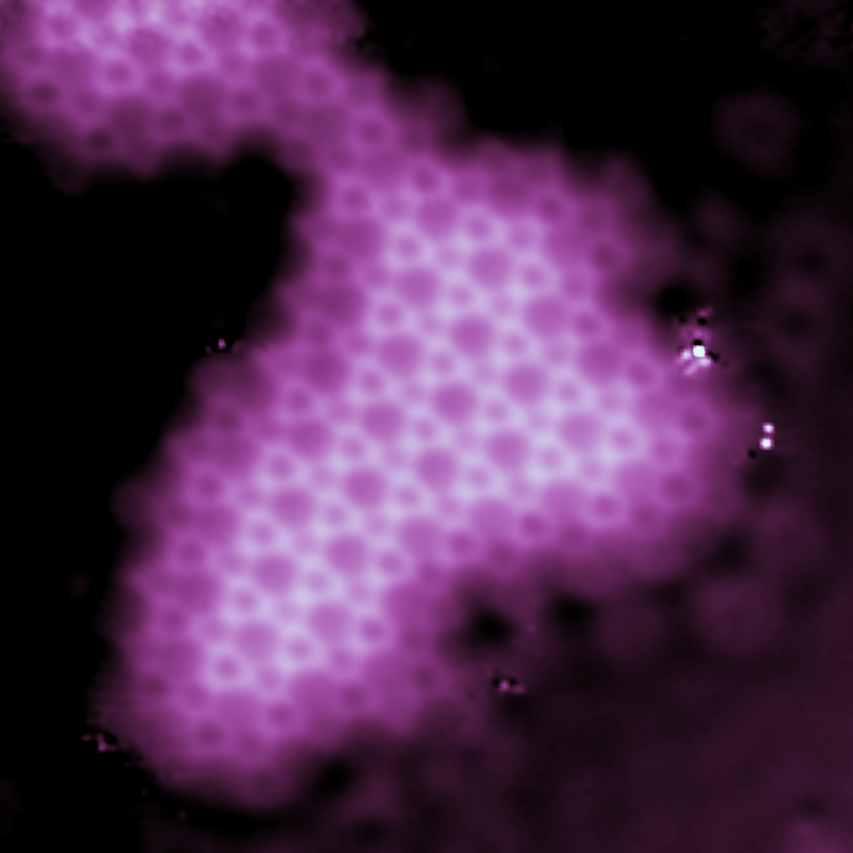

Supplement: Supplementary file 3 — Source data [file 41467_2024_47367_MOESM3_ESM.zip › Supplementary Fig.12/Supplementary Fig.12a/AFM13-4-BrPn-Ag(111)-LHe007 Image LI_Demod_1_X (A).bmp]

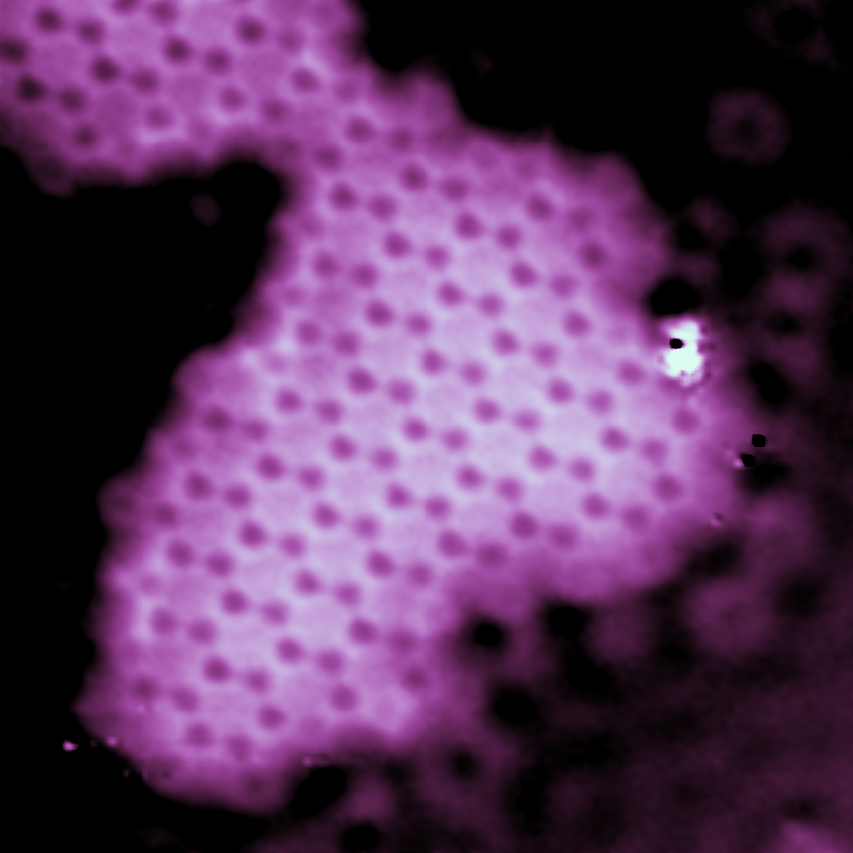

Supplement: Supplementary file 3 — Source data [file 41467_2024_47367_MOESM3_ESM.zip › Supplementary Fig.12/Supplementary Fig.12a/AFM13-4-BrPn-Ag(111)-LHe009 Image LI_Demod_1_X (A)1.3V.bmp]

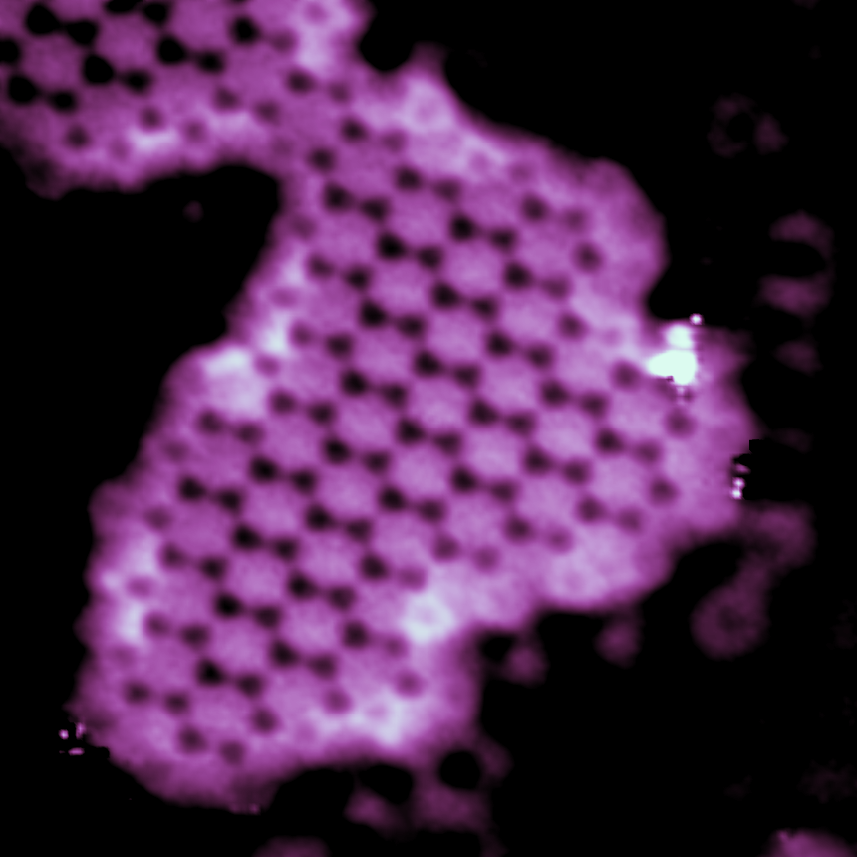

Supplement: Supplementary file 3 — Source data [file 41467_2024_47367_MOESM3_ESM.zip › Supplementary Fig.12/Supplementary Fig.12a/AFM13-4-BrPn-Ag(111)-LHe011 Image LI_Demod_1_X (A)1.2V.bmp]

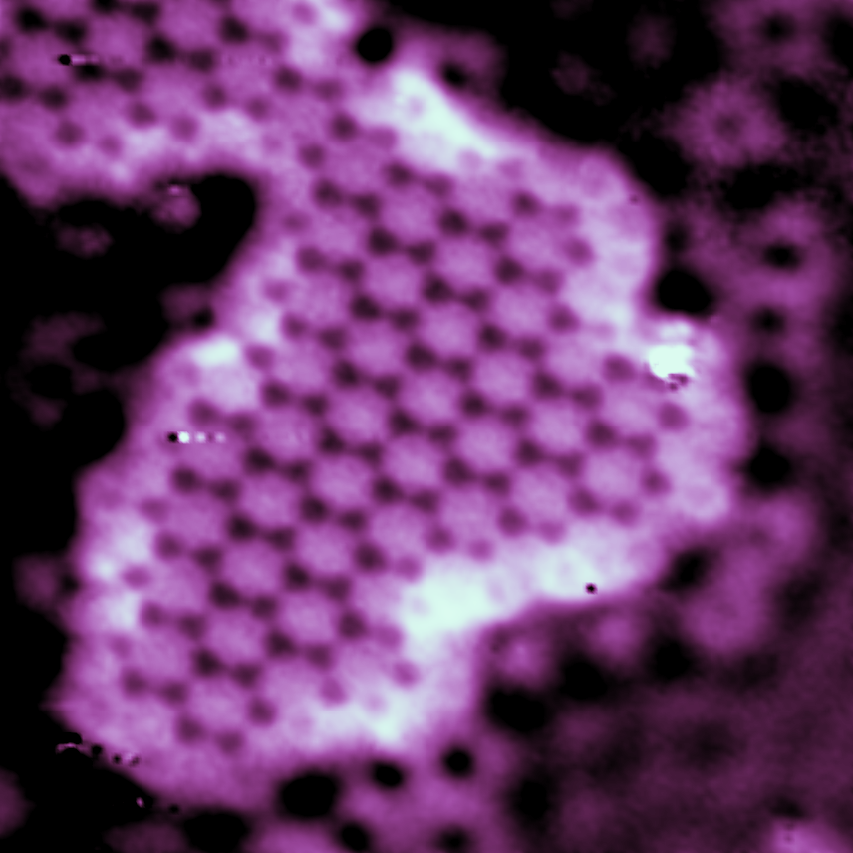

Supplement: Supplementary file 3 — Source data [file 41467_2024_47367_MOESM3_ESM.zip › Supplementary Fig.12/Supplementary Fig.12a/AFM13-4-BrPn-Ag(111)-LHe012 Image LI_Demod_1_X (A)1.1V.bmp]

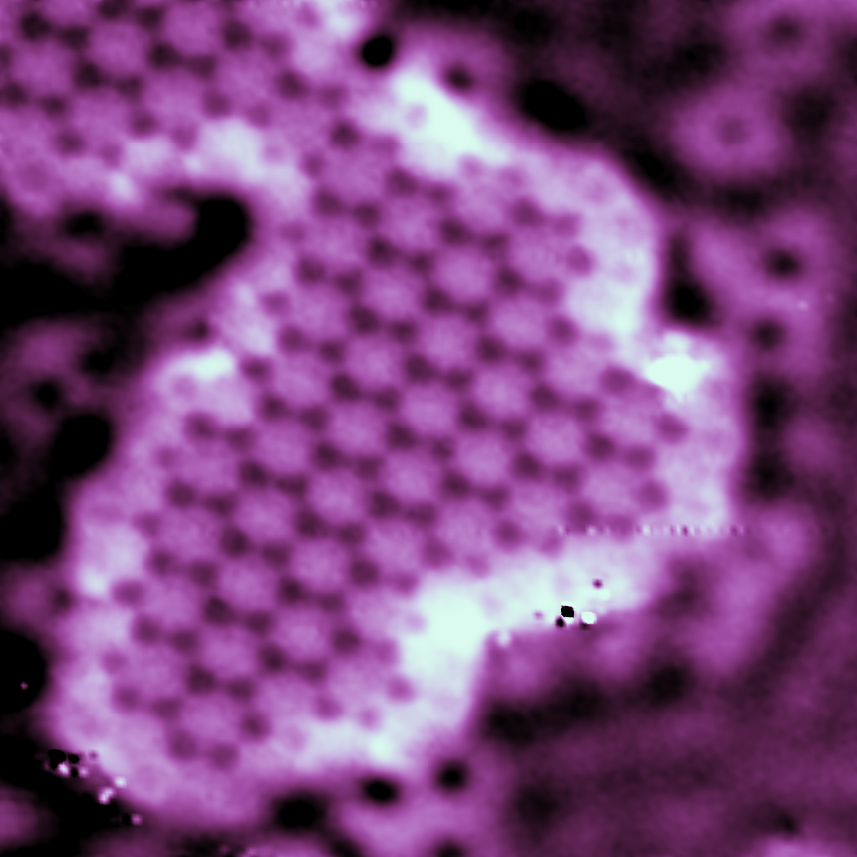

Supplement: Supplementary file 3 — Source data [file 41467_2024_47367_MOESM3_ESM.zip › Supplementary Fig.12/Supplementary Fig.12a/AFM13-4-BrPn-Ag(111)-LHe013 Image LI_Demod_1_X (A)1V.bmp]

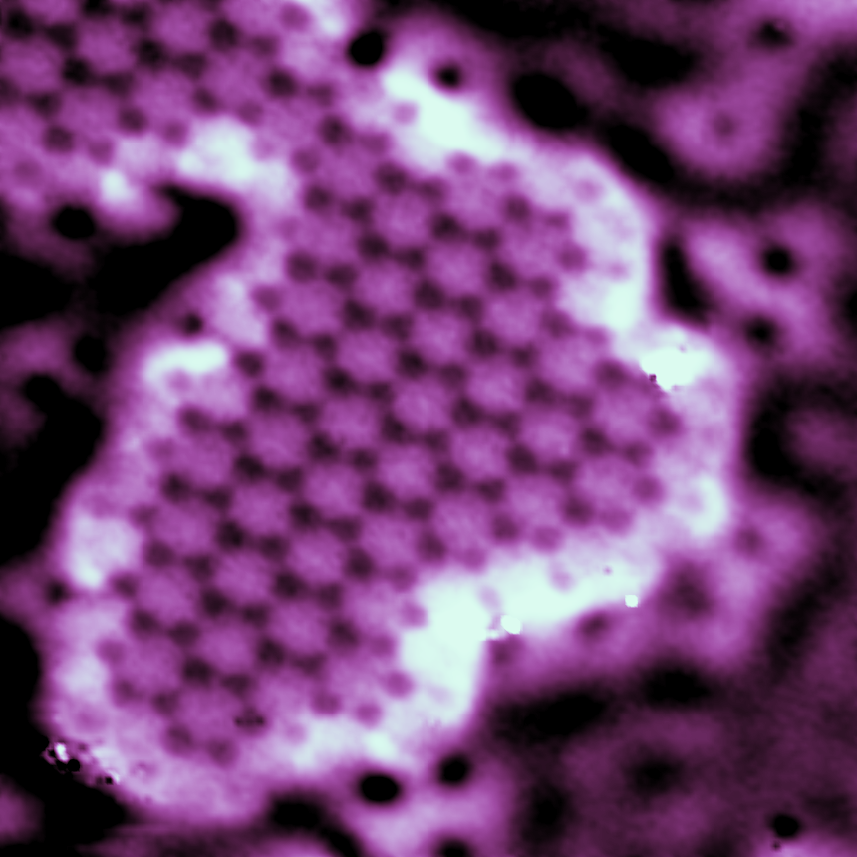

Supplement: Supplementary file 3 — Source data [file 41467_2024_47367_MOESM3_ESM.zip › Supplementary Fig.12/Supplementary Fig.12a/AFM13-4-BrPn-Ag(111)-LHe014 Image LI_Demod_1_X (A)0.9V.bmp]

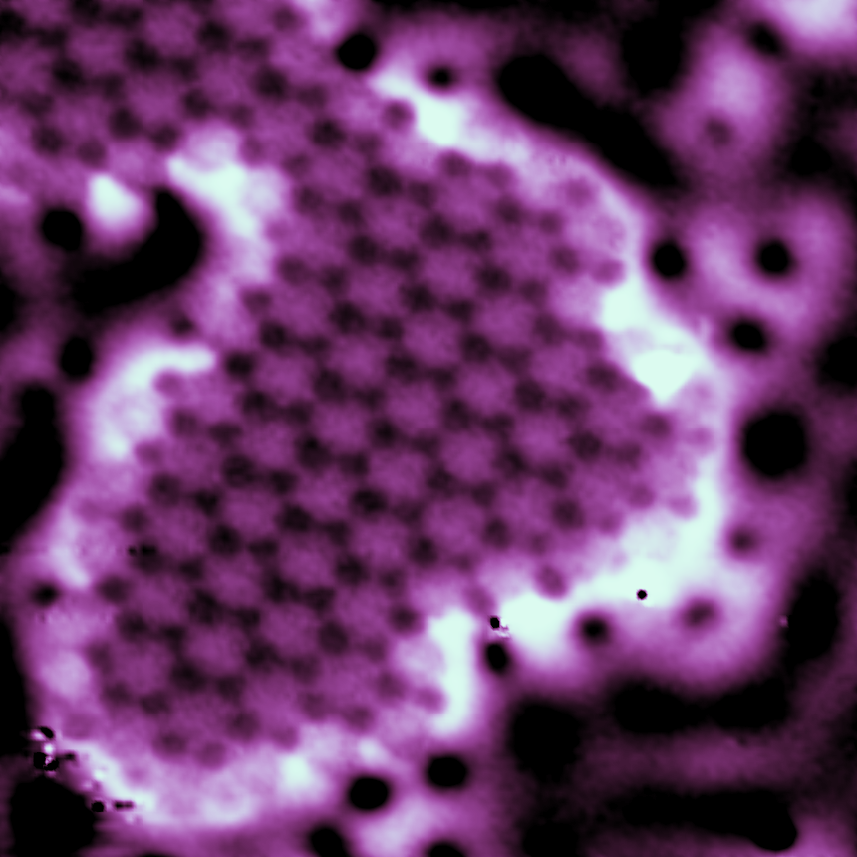

Supplement: Supplementary file 3 — Source data [file 41467_2024_47367_MOESM3_ESM.zip › Supplementary Fig.12/Supplementary Fig.12a/AFM13-4-BrPn-Ag(111)-LHe016 Image LI_Demod_1_X (A)0.7V.bmp]

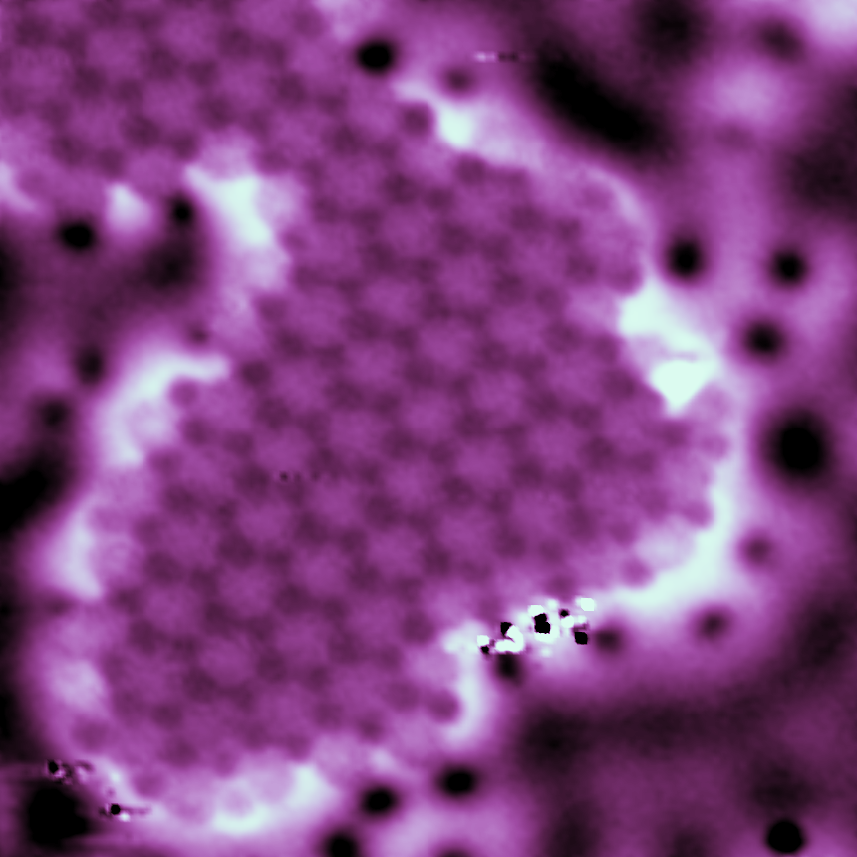

Supplement: Supplementary file 3 — Source data [file 41467_2024_47367_MOESM3_ESM.zip › Supplementary Fig.12/Supplementary Fig.12a/AFM13-4-BrPn-Ag(111)-LHe017 Image LI_Demod_1_X (A)0.6V.bmp]

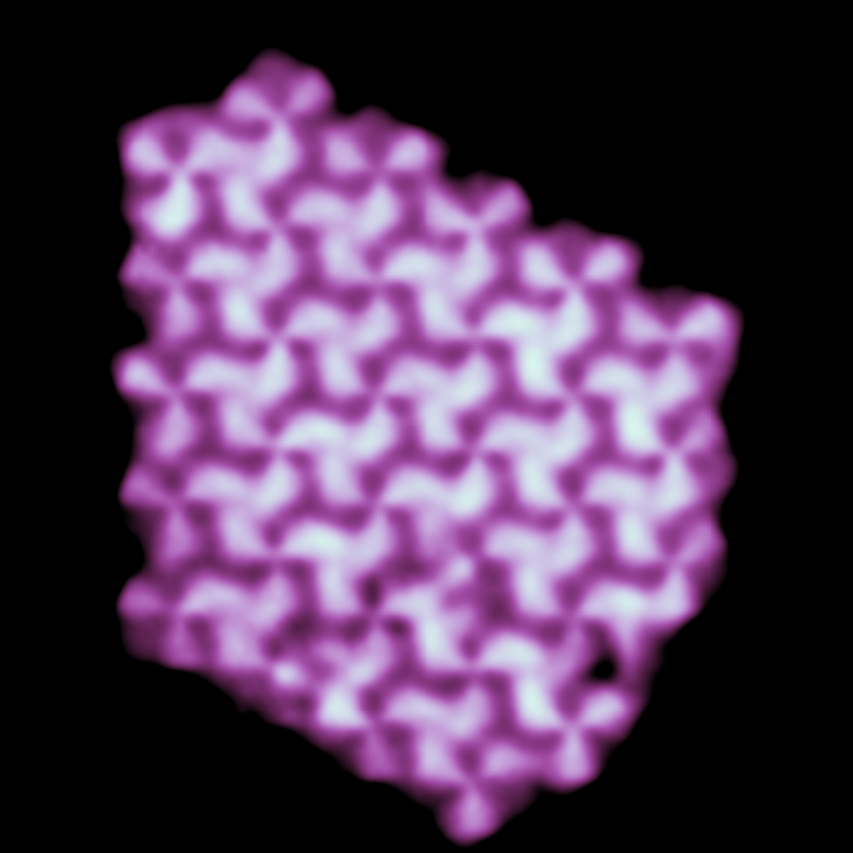

Supplement: Supplementary file 3 — Source data [file 41467_2024_47367_MOESM3_ESM.zip › Supplementary Fig.12/Supplementary Fig.12b/202100627_DBP@Ag(111)_LHe_029 Image Input_7-1.4V.bmp]

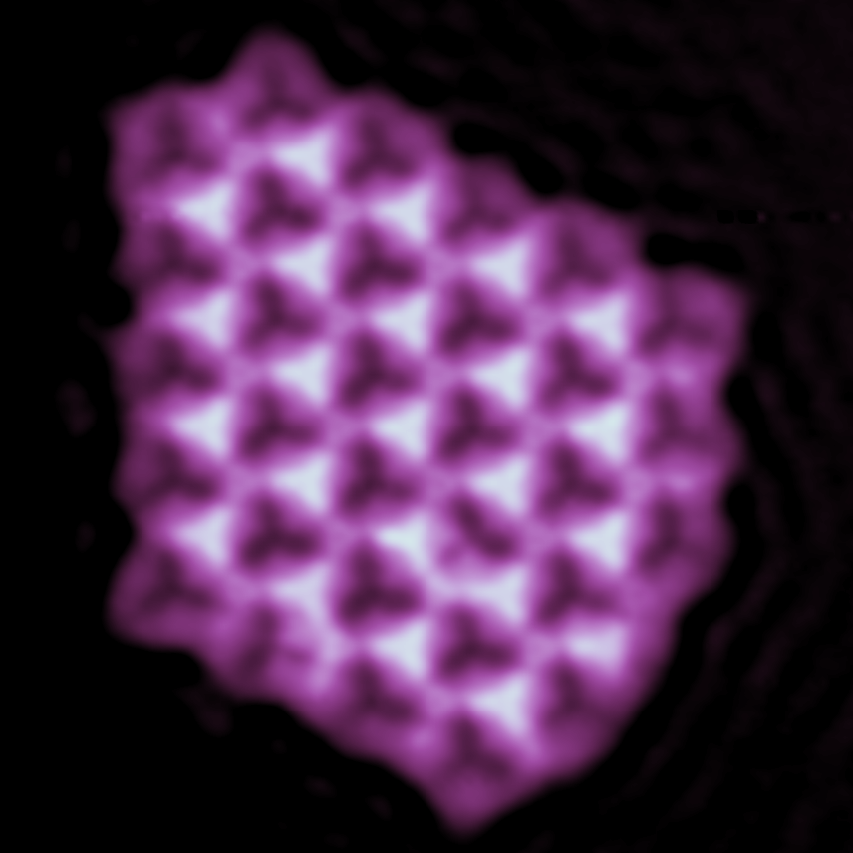

Supplement: Supplementary file 3 — Source data [file 41467_2024_47367_MOESM3_ESM.zip › Supplementary Fig.12/Supplementary Fig.12b/202100627_DBP@Ag(111)_LHe_032 Image Input_7-0.9V.bmp]

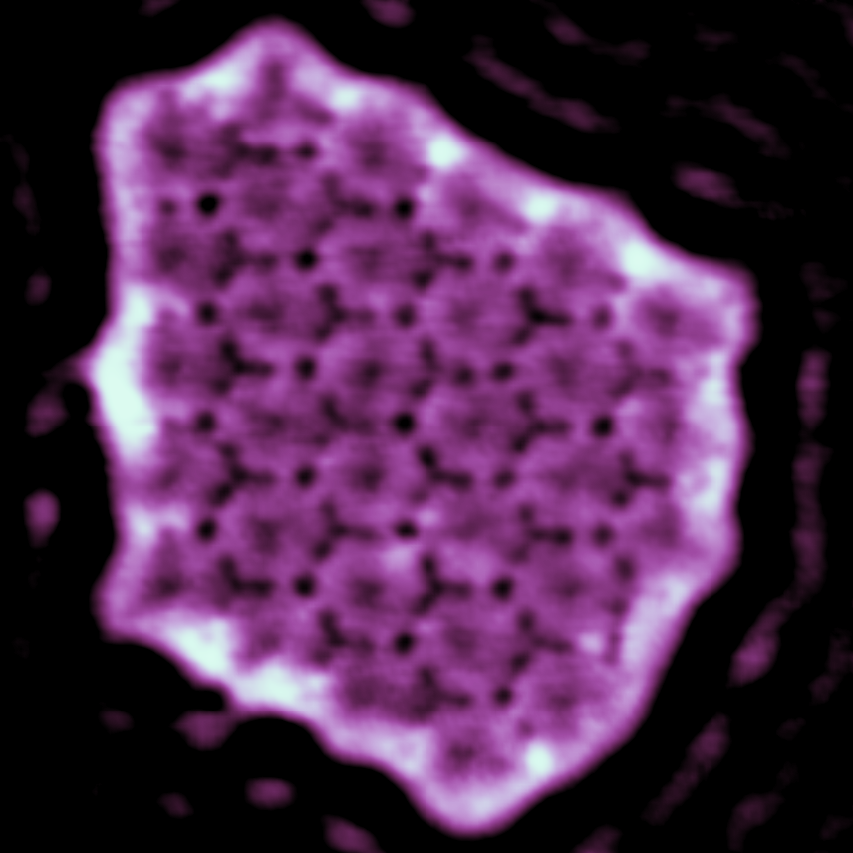

Supplement: Supplementary file 3 — Source data [file 41467_2024_47367_MOESM3_ESM.zip › Supplementary Fig.12/Supplementary Fig.12b/202100627_DBP@Ag(111)_LHe_034 Image Input_7 (V)0.42V.bmp]

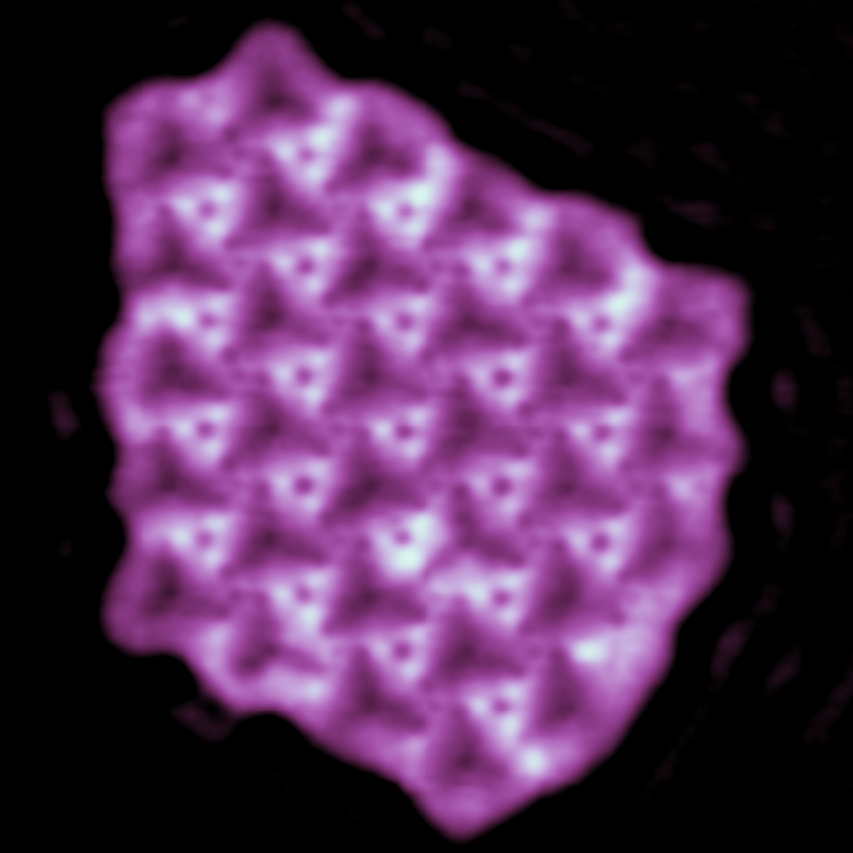

Supplement: Supplementary file 3 — Source data [file 41467_2024_47367_MOESM3_ESM.zip › Supplementary Fig.12/Supplementary Fig.12b/202100627_DBP@Ag(111)_LHe_035 Image Input_7 (V)0.67V.bmp]

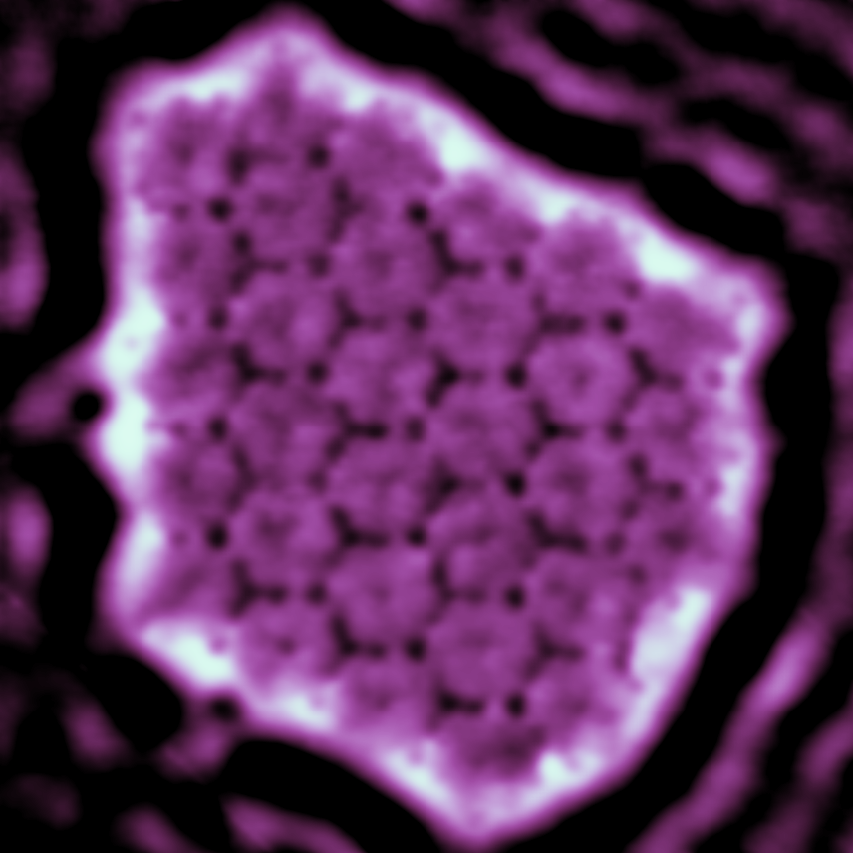

Supplement: Supplementary file 3 — Source data [file 41467_2024_47367_MOESM3_ESM.zip › Supplementary Fig.12/Supplementary Fig.12b/202100627_DBP@Ag(111)_LHe_059 Image Input_7-0.28V.bmp]

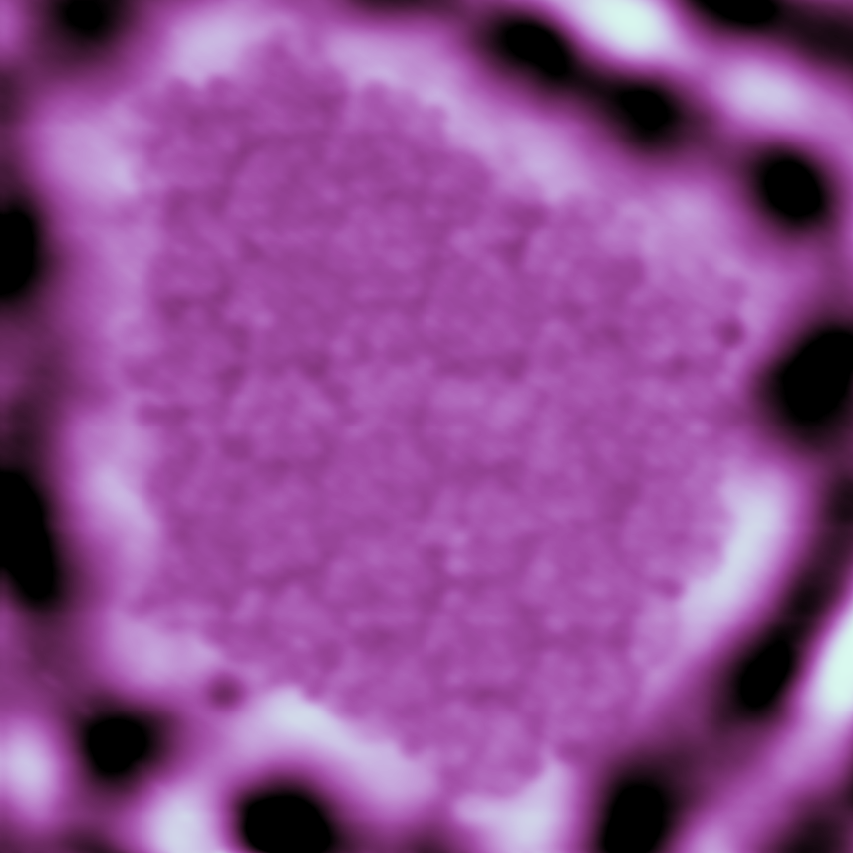

Supplement: Supplementary file 3 — Source data [file 41467_2024_47367_MOESM3_ESM.zip › Supplementary Fig.12/Supplementary Fig.12b/202100627_DBP@Ag(111)_LHe_061 Image Input_7 (V)0.05V.bmp]

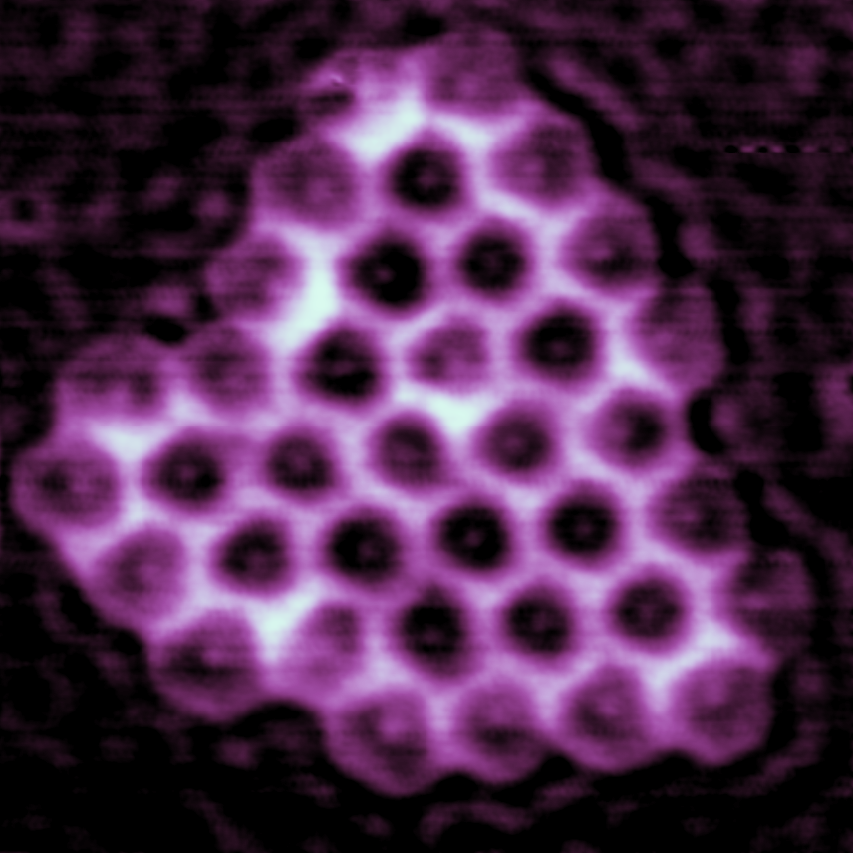

Supplement: Supplementary file 3 — Source data [file 41467_2024_47367_MOESM3_ESM.zip › Supplementary Fig.12/Supplementary Fig.12c/20211203_DBP@Au(111)_LHe_031 Image Input_7 (V)-0.87V.bmp]

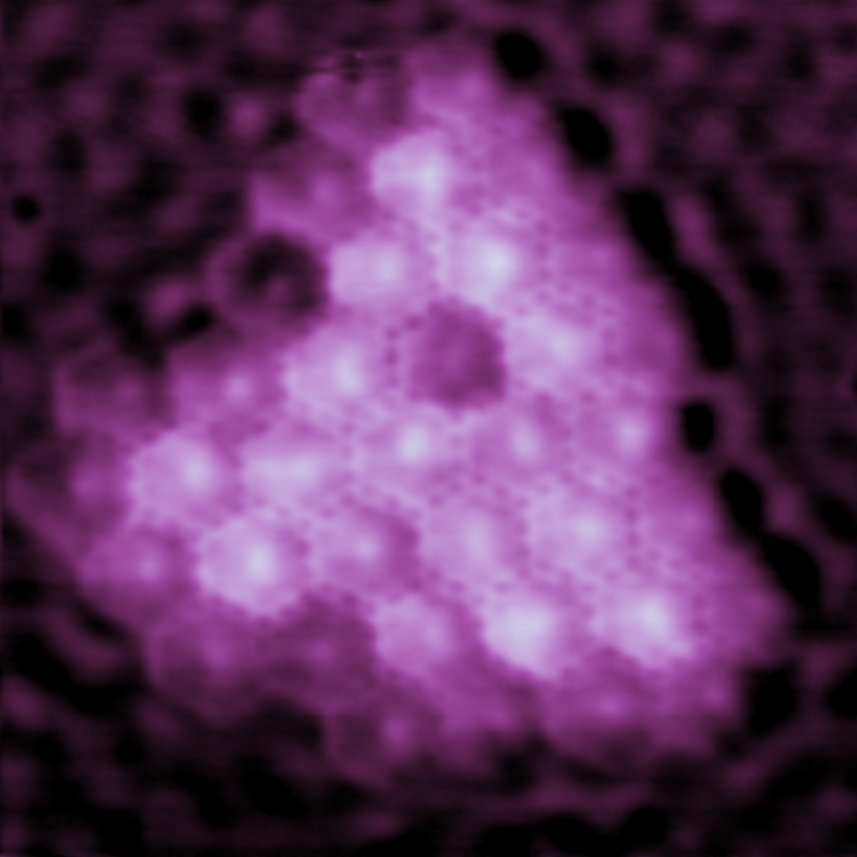

Supplement: Supplementary file 3 — Source data [file 41467_2024_47367_MOESM3_ESM.zip › Supplementary Fig.12/Supplementary Fig.12c/20211203_DBP@Au(111)_LHe_033 Image Input_7 (V)0.37V.bmp]

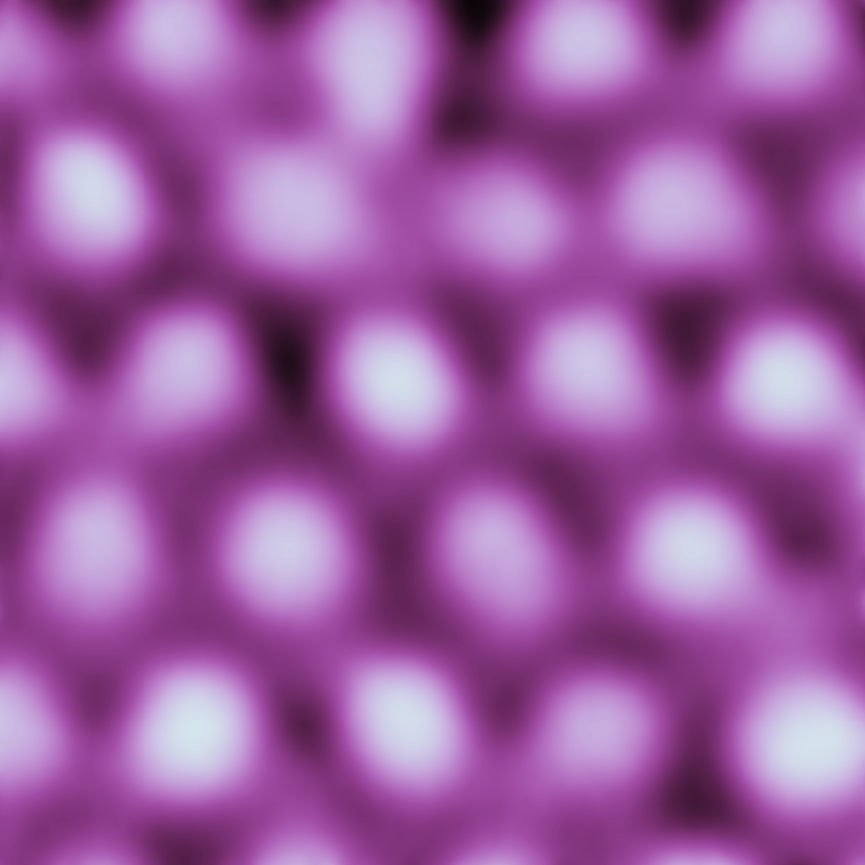

Supplement: Supplementary file 3 — Source data [file 41467_2024_47367_MOESM3_ESM.zip › Supplementary Fig.3/Supplementary Fig.3b/202301223_LHe_Agú¿111ú⌐_4BrPn_0156 Image Input_7 (V).stp202301223_LHe_Agú¿111ú⌐_4BrPn_0156 Image Input_7 (V).bmp]

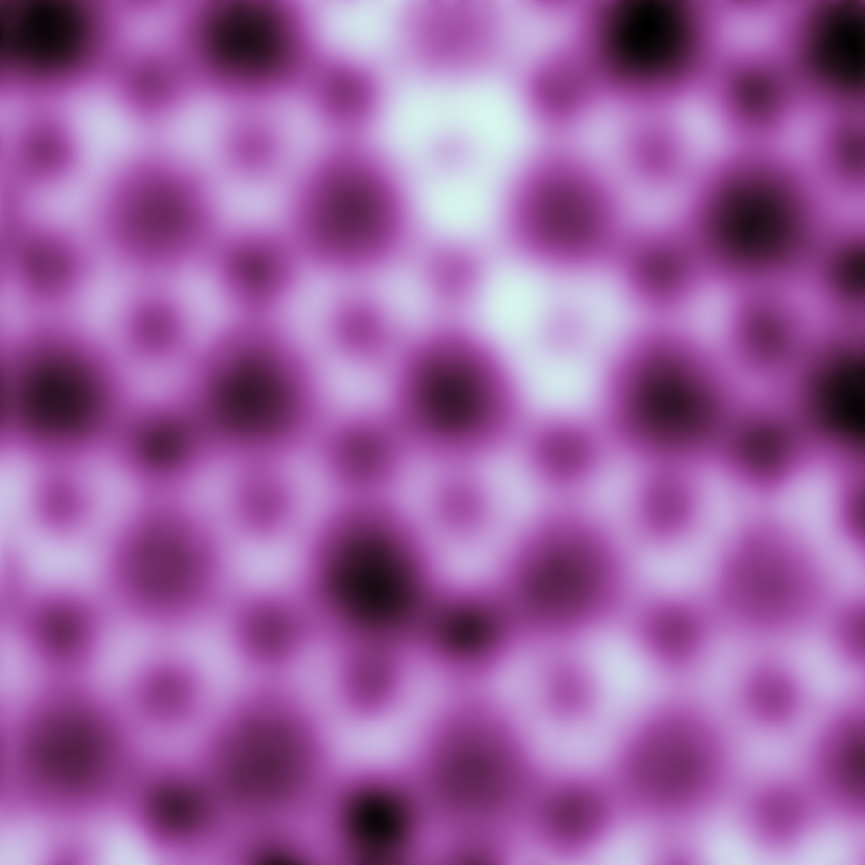

Supplement: Supplementary file 3 — Source data [file 41467_2024_47367_MOESM3_ESM.zip › Supplementary Fig.3/Supplementary Fig.3d/202301223_LHe_Agú¿111ú⌐_4BrPn_0142 Image Input_7 (V).stp202301223_LHe_Agú¿111ú⌐_4BrPn_0142 Image Input_7 (V).bmp]

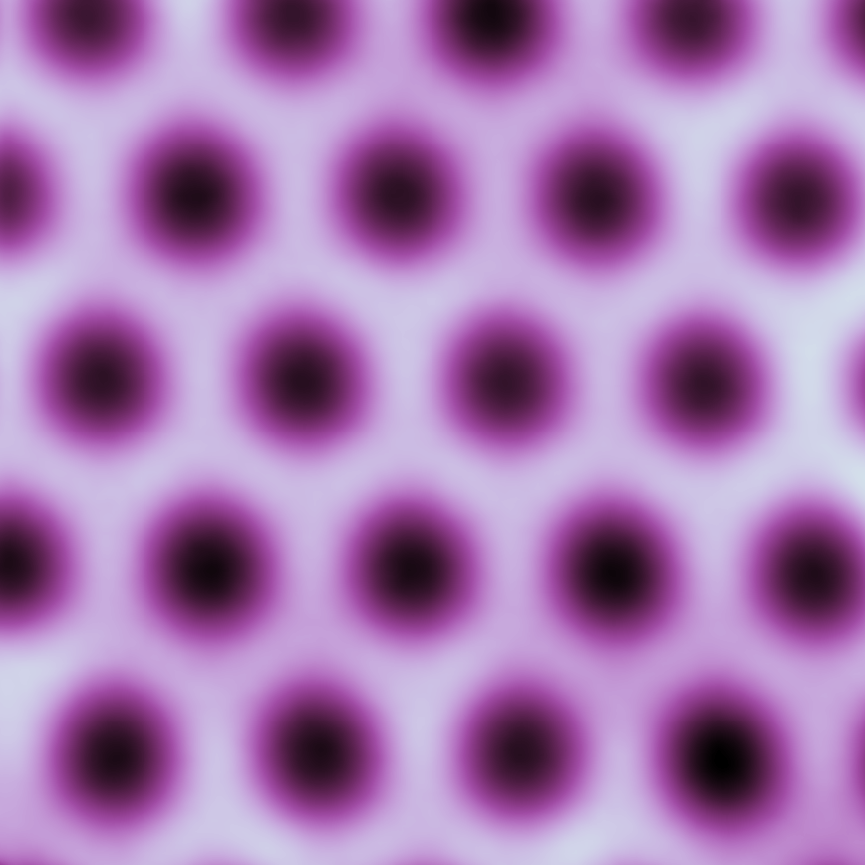

Supplement: Supplementary file 3 — Source data [file 41467_2024_47367_MOESM3_ESM.zip › Supplementary Fig.3/Supplementary Fig.3f/202301223_LHe_Agú¿111ú⌐_4BrPn_0137 Image Input_7 (V).stp202301223_LHe_Agú¿111ú⌐_4BrPn_0137 Image Input_7 (V).bmp]

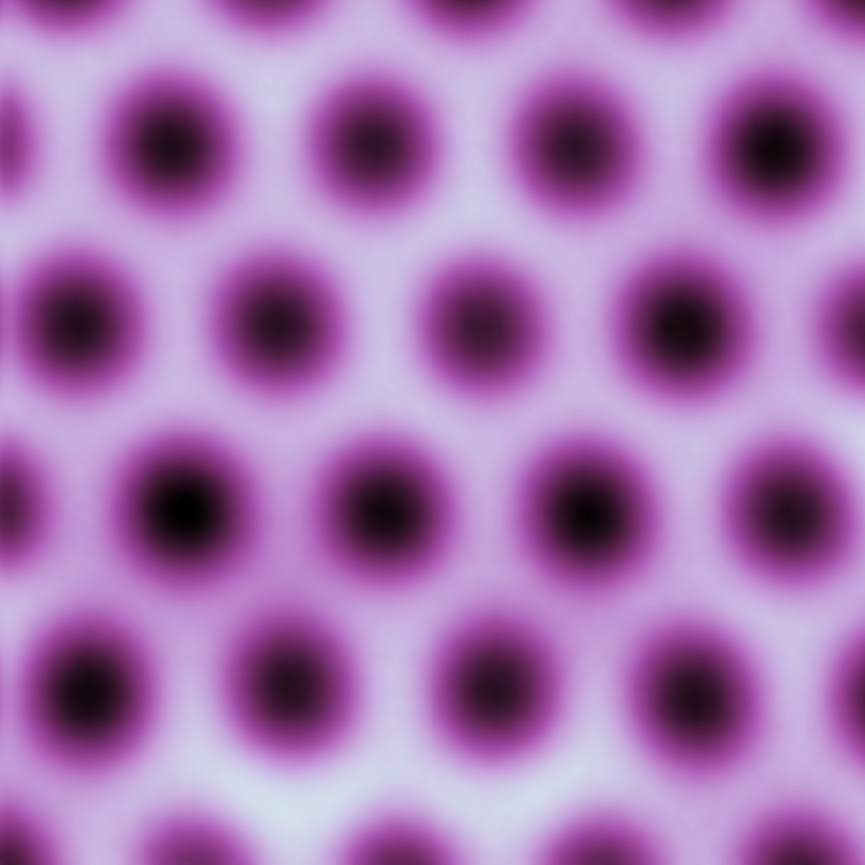

Supplement: Supplementary file 3 — Source data [file 41467_2024_47367_MOESM3_ESM.zip › Supplementary Fig.3/Supplementary Fig.3g/202301223_LHe_Agú¿111ú⌐_4BrPn_0139 Image Input_7 (V).stp202301223_LHe_Agú¿111ú⌐_4BrPn_0139 Image Input_7 (V).bmp]

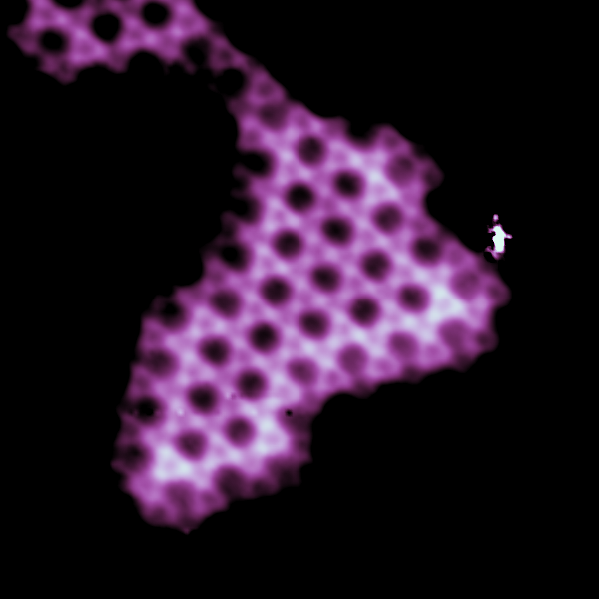

Supplement: Supplementary file 3 — Source data [file 41467_2024_47367_MOESM3_ESM.zip › Supplementary Fig.5/Supplementary Fig.5d/AFM13-4-BrPn-Ag(111)-LHe006 Image LI_Demod_1_X (A)1.bmp]

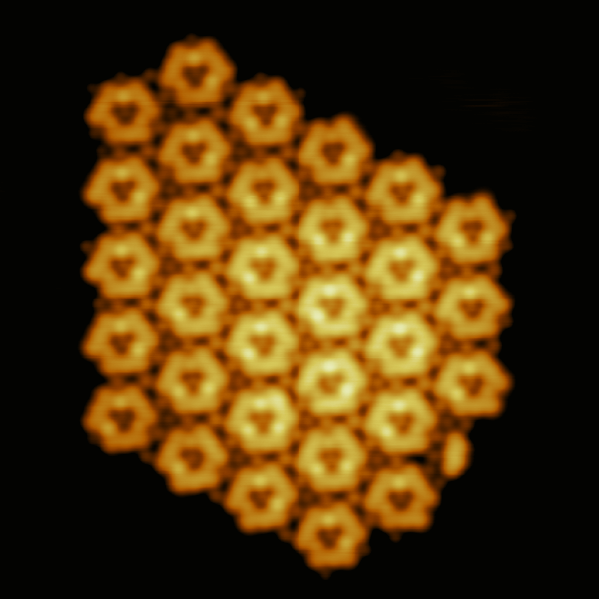

Supplement: Supplementary file 3 — Source data [file 41467_2024_47367_MOESM3_ESM.zip › Supplementary Fig.6/Supplementary Fig.6a/202100627_DBP@Ag(111)_LHe_058 Image Z1 (m).bmp]

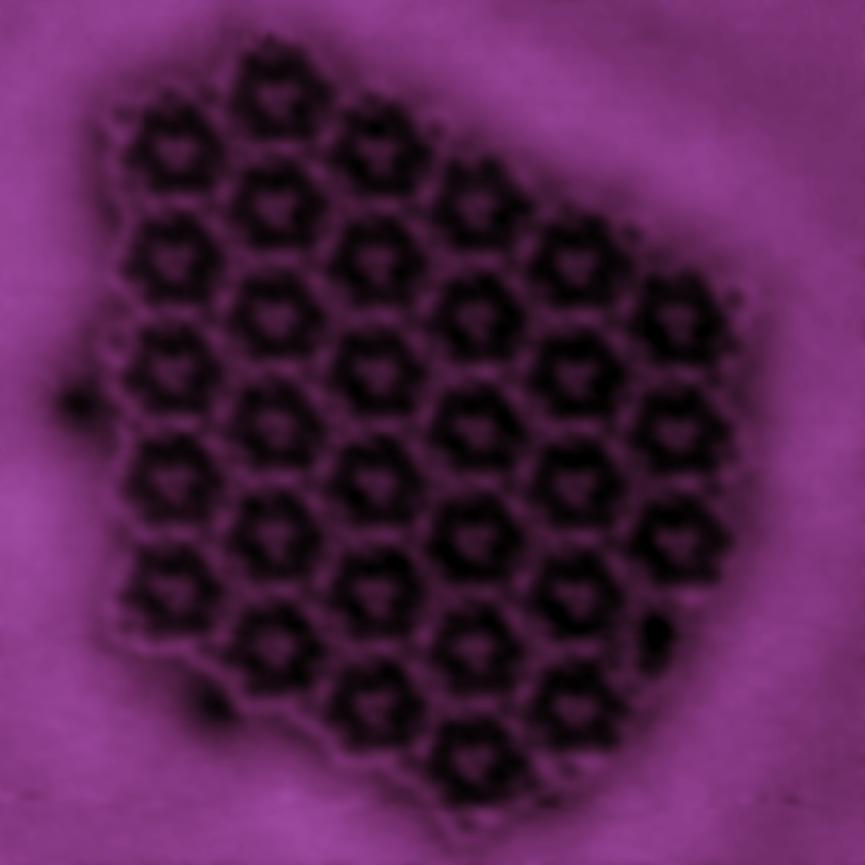

Supplement: Supplementary file 3 — Source data [file 41467_2024_47367_MOESM3_ESM.zip › Supplementary Fig.6/Supplementary Fig.6c/202100627_dbp@ag(111)_lhe_036 image input_736 image input_7 (v).bmp]

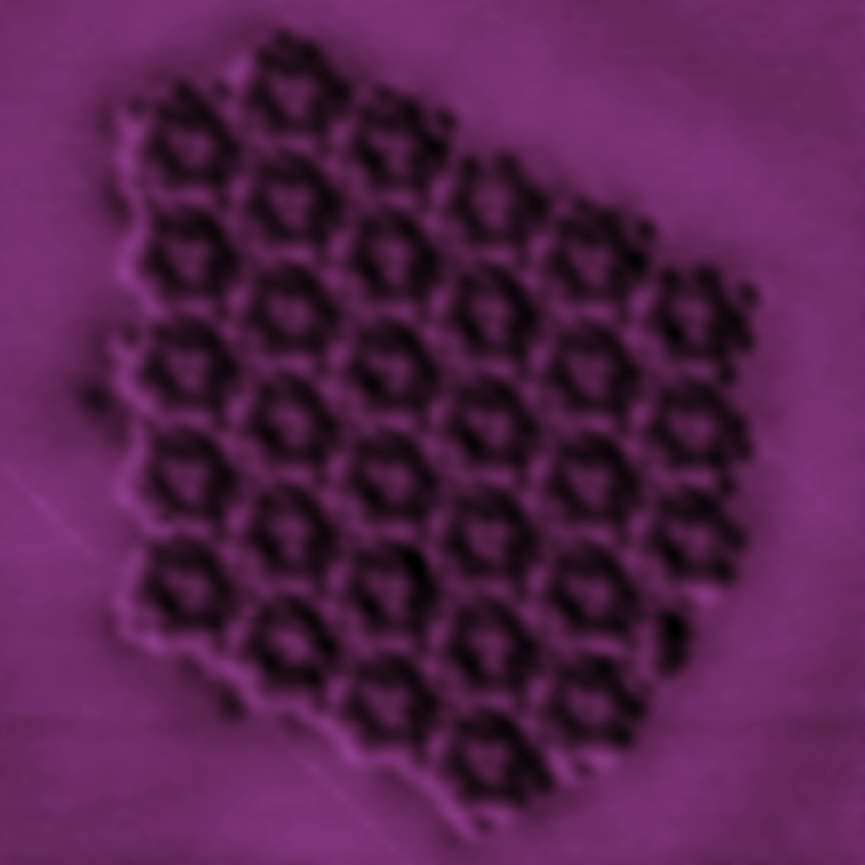

Supplement: Supplementary file 3 — Source data [file 41467_2024_47367_MOESM3_ESM.zip › Supplementary Fig.6/Supplementary Fig.6d/202100627_DBP@Ag(111)_LHe_063 Image Input_7.bmp]

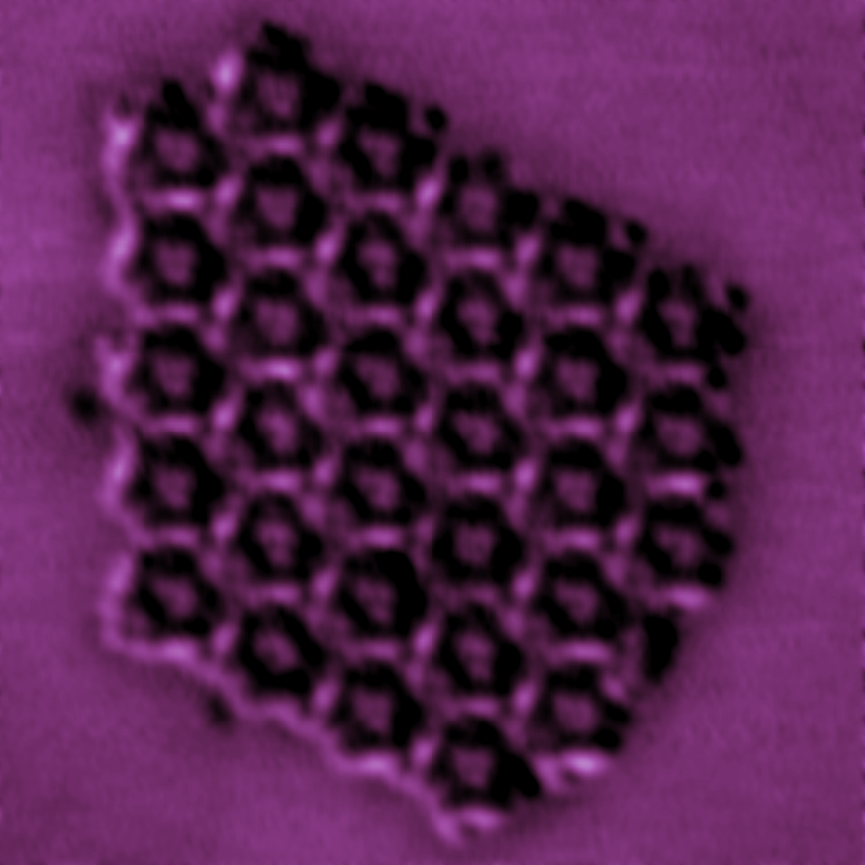

Supplement: Supplementary file 3 — Source data [file 41467_2024_47367_MOESM3_ESM.zip › Supplementary Fig.6/Supplementary Fig.6e/202100627_DBP@Ag(111)_LHe_040 Image Input_7.bmp]

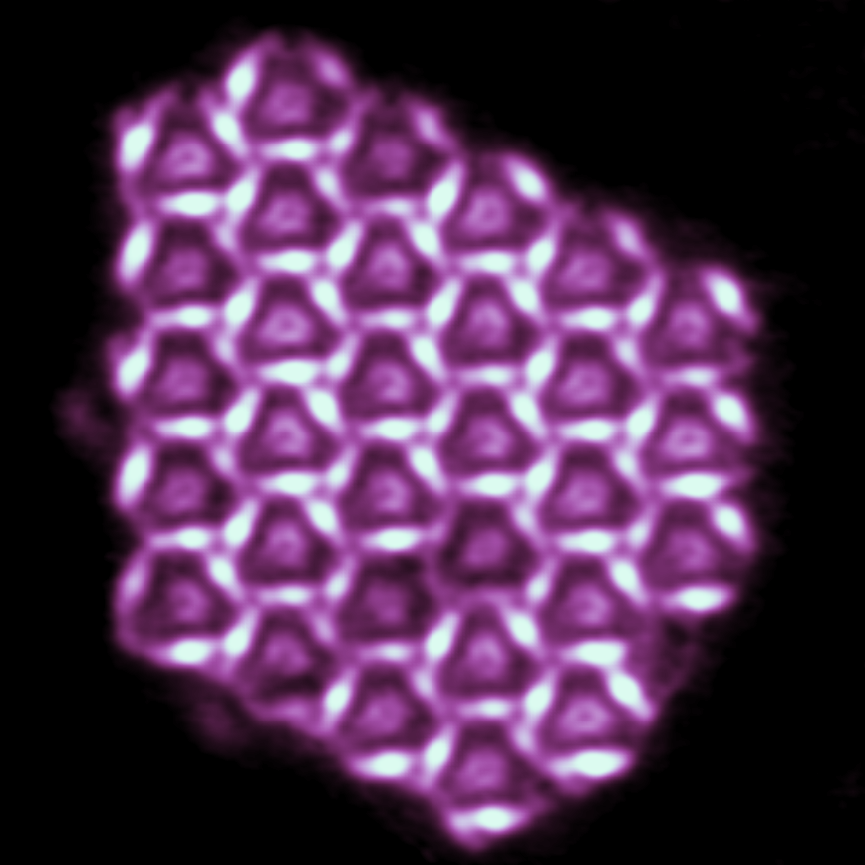

Supplement: Supplementary file 3 — Source data [file 41467_2024_47367_MOESM3_ESM.zip › Supplementary Fig.6/Supplementary Fig.6f/202100627_DBP@Ag(111)_LHe_064 Image Input_7 (V).bmp]

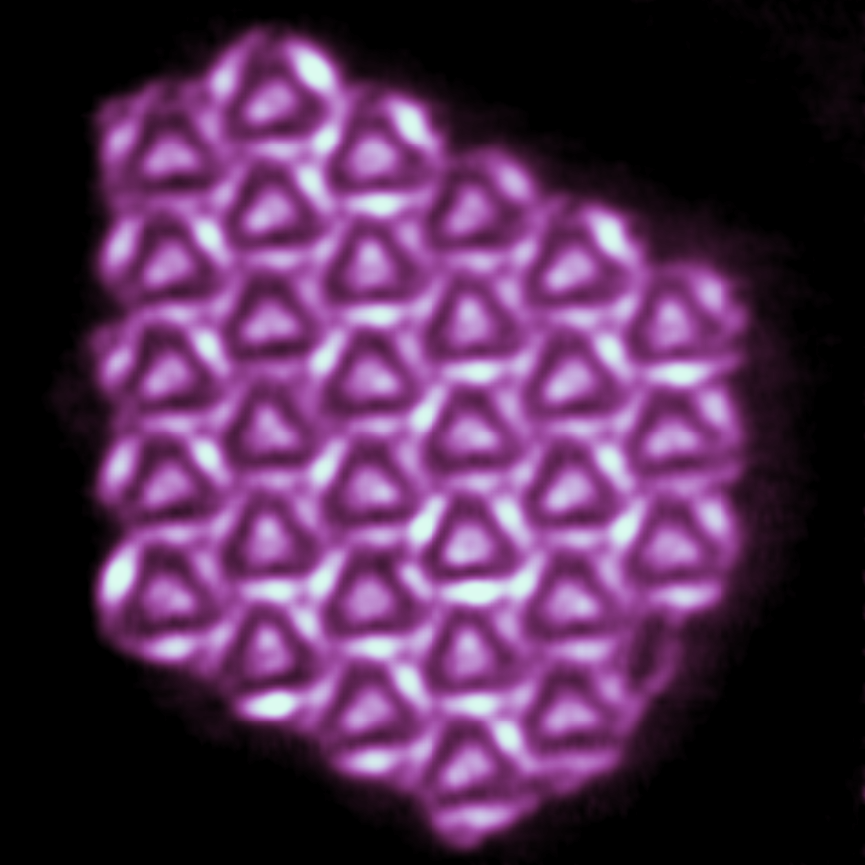

Supplement: Supplementary file 3 — Source data [file 41467_2024_47367_MOESM3_ESM.zip › Supplementary Fig.6/Supplementary Fig.6g/202100627_DBP@Ag(111)_LHe_041 Image Input_7.bmp]
